# Genomic insights into the plasmidome of non-tuberculous mycobacteria

**DOI:** 10.1186/s13073-025-01443-7

**Published:** 2025-03-04

**Authors:** Margo Diricks, Florian P. Maurer, Viola Dreyer, Ivan Barilar, Christian Utpatel, Matthias Merker, Nils Wetzstein, Stefan Niemann

**Affiliations:** 1 Molecular and Experimental Mycobacteriology, Research Center Borstel, Borstel, Germany; 2 German Center for Infection Research (DZIF), Partner Site Hamburg-Lübeck-Borstel-Riems, Borstel, Germany; 3 National and WHO Supranational Reference Laboratory for Mycobacteria, Research Center Borstel, Leibniz Lung Center, Borstel, Germany; 4 Institute of Medical Microbiology, Virology and Hygiene, University Medical Center Hamburg-Eppendorf, Hamburg, Germany; 5 Evolution of the Resistome, Research Center Borstel, Borstel, Germany; 6 Department of Internal Medicine, Infectious Diseases, Goethe University Frankfurt, University Hospital, Frankfurt Am Main, Germany; 7 Mycobacterial Infection Research Unit (MIRU), Goethe University Frankfurt, Frankfurt Am Main, Germany

**Keywords:** Non-tuberculous mycobacteria, Plasmids, Genomics, Antimicrobial resistance

## Abstract

**Background:**

Non-tuberculous mycobacteria (NTM) are a diverse group of environmental bacteria that are increasingly associated with human infections and difficult to treat. Plasmids, which might carry resistance and virulence factors, remain largely unexplored in NTM.

**Methods:**

We used publicly available complete genome sequence data of 328 NTM isolates belonging to 125 species to study gene content, genomic diversity, and clusters of 196 annotated NTM plasmids. Furthermore, we analyzed 3755 draft genome assemblies from over 200 NTM species and 5415 short-read sequence datasets from six clinically relevant NTM species or complexes including *M. abscessus*, *M. avium* complex, *M. ulcerans* complex and *M. kansasii* complex, for the presence of these plasmids.

**Results:**

Between one and five plasmids were present in approximately one-third of the complete NTM genomes. The annotated plasmids varied widely in length (most between 10 and 400 kbp) and gene content, with many genes having an unknown function. Predicted gene functions primarily involved plasmid replication, segregation, maintenance, and mobility. Only a few plasmids contained predicted genes that are known to confer resistance to antibiotics commonly used to treat NTM infections. Out of 196 annotated plasmid sequences, 116 could be grouped into 31 clusters of closely related sequences, and about one-third were found across multiple NTM species. Among clinically relevant NTM, the presence of NTM plasmids showed significant variation between species, within (sub)species, and even among strains within (sub)lineages, such as dominant circulating clones of *Mycobacterium abscessus.*

**Conclusions:**

Our analysis demonstrates that plasmids are a diverse and heterogeneously distributed feature in NTM bacteria. The frequent occurrence of closely related putative plasmid sequences across different NTM species suggests they may play a significant role in NTM evolution through horizontal gene transfer at least in some groups of NTM. However, further in vitro investigations and access to more complete genomes are necessary to validate our findings, elucidate gene functions, identify novel plasmids, and comprehensively assess the role of plasmids in NTM.

**Supplementary Information:**

The online version contains supplementary material available at 10.1186/s13073-025-01443-7.

## Background

Non-tuberculous mycobacteria (NTM) refer to all *Mycobacterium* species—over 200 in total—excluding *M. tuberculosis,* the causative agent of tuberculosis, and *M. leprae*, responsible for leprosy. NTM are environmental organisms ubiquitously found in soil and water. Some species act as opportunistic pathogens that can cause severe disease especially in immunocompromised patients or those with structural lung disease such as cystic fibrosis [1, 2]. NTM infections often affect the lungs but can also involve other parts of the human body, such as the skin, bones, lymph nodes, and bloodstream [3]. The diagnosis and treatment of NTM infections is challenging due to their slow growth and the limited number of effective antibiotics [4]. Recent data suggests that NTM infections are increasing in frequency globally [5]. Therefore, NTM are considered a growing public health concern, especially in countries with low tuberculosis incidence.

Plasmids can play a pivotal role in the emergence and evolution of human pathogens, for example via their contribution to the acquisition of antibiotic resistance [6] or virulence genes [7]. While plasmids are absent in the human pathogens *M. tuberculosis* and *M. leprae*, they have been reported in several clinically relevant NTM. For instance, *M. ulcerans* virulence is related to a large plasmid (e.g., pMUM001) that encodes the exotoxin mycolactone. This toxin induces apoptosis in a wide variety of cells, leading to necrotic skin lesions that are the hallmark of Buruli ulcer disease [8–10]. In a *M. avium* complex strain, the presence of plasmids was associated with high mortality and a progressive increase in bacterial load in mice [11]. In addition, the *M. avium* plasmid pMAH135 is suggested to be involved in pathogenicity and progressive human pulmonary disease [12, 13]. A clonal *M. abscessus* subsp. *massiliense* strain (BRA100) that was associated with a nationwide epidemic of surgical infections in Brazil, carried a putative plasmid (pMAB02) [14, 15] which contained an Esx gene cluster coding for a mycobacterial type VII secretion system potentially important for conjugation [16]. Some strains additionally carried another plasmid (pMAB01 or pBRA100), which belonged to the broad-host-range plasmid subgroup IncP-1β and encodes resistance genes against mercury, aminoglycosides, and other drugs [14–16]. Mercury and copper resistance genes have also been identified in plasmid DNA from *M. abscessus*, *M. marinum*, and *M. scrofulaceum* [17–20]. Lastly, the global *M. intracellulare* subsp*. chimaera* outbreak strain Zuerich-1, which is linked to cardiac surgery-related infections through contaminated heater-cooler units, harbors five plasmids but their role in pathogenicity or transmission is currently unknown [21, 22].

It has been suggested that plasmids can transfer between mycobacterial species. Rabello and colleagues demonstrated with in vitro mating experiments that the plasmid pMA100 from *M. avium* could be transferred to *M. kansasii* and *M. bovis*, but not to *M. smegmatis* [23]*.* In addition, in silico studies have found closely related plasmid sequences in different NTM species [24, 25].

Although plasmids may play a crucial role in adaptation and survival in different environments, and potentially contribute to virulence and antibiotic resistance, the plasmidome of non-tuberculous mycobacteria remains poorly understood and under-researched. To address this, we determined general characteristics, gene content, evolutionary relationships, and distribution of NTM plasmids using more than 9000 genomic datasets from more than 200 NTM species using in silico approaches.

## Methods

### Data

All available completely assembled genomes of *Mycobacteriaceae* (taxid 1762) were downloaded from the RefSeq database [26] using NCBI Datasets CLI (June 26th, 2023). Strains belonging to the *M. tuberculosis* complex were manually excluded. Strains with identical names or a pairwise mash distance of 0 (calculated using Mashtree [27] v1.2.0) were identified, and only the most recent genome was retained. For the remaining 328 NTM genomes, a sequence report was generated, and all associated assembly and annotation files were downloaded. The final dataset comprised 98 plasmid-carrying genomes with a total of 196 sequences annotated as plasmids and 230 plasmid-free genomes (Additional file 1: Table S1 and S2).

In addition, all NTM incomplete draft assemblies (contig and scaffold level) in NCBI were downloaded on November 28th, 2024. Following assemblies were removed: *M. tuberculosis*, *M. leprae*, and *M. lepromatosis*, as well as assemblies with inconclusive, failed, or uncultured ANI check status (as annotated in NCBI), leaving a final set of 3755 NTM draft genomes (Additional file 1: Table S1). Lastly, 5415 Illumina short-read sequencing datasets from clinically relevant NTM were downloaded from SRA (Additional file 1: Table S3). For *M. abscessus*, these comprised a diverse set of 1486 sequences selected from Diricks et al. [28]. For *M. avium* complex (MAC), we used 1798 sequences analyzed in Wetzstein et al. [25, 29], complemented with a curated set of 469 M*. avium* subsp. *paratuberculosis*, *avium*, and *silvaticum* isolates from Mizzi et al. [30] as well as 184 additional isolates related to the HCU outbreak [21]. For *M. ulcerans* and *M. kansasii* complex, we downloaded all available Illumina data from SRA and removed sequences with low coverage (< 30 ×), low quality or contamination resulting in 983 M*. ulcerans* complex isolates and 495 M*. kansasii* complex isolates (Additional file 1: Table S3).

### Construction of gene families

Protein-coding genes were extracted from all complete assemblies and clustered into protein families based on amino acid sequence similarity. For that, reciprocal best hits (RBHs) among all contigs were identified using the easy-RBH module of MMSeqs [31] v14.7 with a threshold of *E*-value ≤ 1 × 10^−10^. The RBH pairs were then globally aligned with Parasail-python (v1.3.4 using the Needleman-Wunsch algorithm). RBH pairs with a global amino acid sequence identity of 30% were used as input for clustering into protein families using the Markov cluster algorithm (MCL) [32, 33] with an inflation parameter of 2.

### Phylogenetic reconstruction

Protein sequences of each protein family were aligned using MAFFT [34] v7.520 with the L-INS-i algorithm. The species tree comprising complete genomes was reconstructed using IQ-TREE [35] v2.2.2.7 from the alignment of all universal single-copy families with a partitioned alignment and amino acid substitution model parameters “-mset LG -madd LG4X,” while accounting for the variable evolutionary rate of each family (-p). The trees were inferred with 1000 bootstrap replicates. The phylogenetic tree was rooted using the branch leading to *M. abscessus* and *M. chelonae* as they have been shown to be the most ancestral [1]. To reconstruct species(complex)-specific trees, short reads were first assembled with shovill [36] v.1.1.0 using skesa [37] or spades [38] as assembly algorithm and Mashtree [27] v.1.2.0 was used to infer whole genome clustering. The resulting trees were rooted at the midpoint. All trees were visualized and annotated using iTOL [39].

### In silico prediction of incompatibility, mobility, plasmid genes, and plasmid-borne contigs

The incompatibility group (determined via PlasmidFinder [40]), plasmid multi-locus sequence typing group (pMLST), topology (linear/circular) and putative replicon, relaxase, and mobility genes were extracted from metadata stored in the curated plasmid database PLSDB (https://ccb-microbe.cs.uni-saarland.de/plsdb2025) [41, 42] (Additional file 1: Table S2). In addition, platon [43] v1.6 (database v.1.5.0) and PLASMe [44] v1.1 with default values were used to identify plasmid genes and/or plasmid-borne contigs in complete and draft genomes (Additional file 1: Table S2).

### Detection of resistance, stress, and virulence genes

AMRfinderPlus v.3.11.2 (gene database version 2023–09–26.1) was used to detect putative resistance, stress, and virulence genes with default values (strict hits) and with relaxed thresholds of 30% amino acid sequence identity and 70% genome coverage (loose hits) to identify more diverged homologues [45]. In addition, abricate [46] v.1.0.1 was used with default values in combination with the virulence factor database (vfdb version 2024–05–07) [47] to screen for additional virulence genes not included in the AMRfinderPlus database.

### Clustering of homologous plasmids

Pairwise mash distances, calculated using mashtree v.1.2.0 [27], were used as an inverse measure of whole-plasmid similarity, with greater mash distances indicating lower sequence similarity. The resulting distance matrix was used as input for the assignClones function of the polysat R package to classify plasmids into clusters with a threshold of 0.05 similar to previous studies [41, 48, 49]. As a result, plasmids within the same cluster have a mash distance of less than 0.05 (corresponding to more than 95% average nucleotide identity, ANI) with at least one other plasmid in that cluster. The ANI and alignment fraction between plasmids from NTM and other bacteria was assessed with fastANI [50] v.1.33 –fragLen 500 -t 16 as described previously for plasmids [51].

### Prediction of plasmid presence starting from raw sequence reads or draft genomes

We searched for the presence of 111 annotated NTM plasmid sequences from complete genomes (1 per cluster) in 5415 short-read sequence datasets and 3755 draft genomes. In addition, we also searched for the plasmid from the Brazilian outbreak strain (pBRA100) for which the plasmid sequence was available but no chromosomal sequence (i.e., a complete genome was lacking for this host) (Additional file1: Table S2). For raw sequence reads, we used our custom pipeline NTMseq [52] which includes SRST2 [53] v.0.2.0 for plasmid sequence detection starting from short read sequencing data. FastANI [50] v.1.33 with a fragment length of 500 and 3000 was used for plasmid sequence detection in draft assemblies (using a cut-off of > 95% ANI and > 90% alignment fraction).

## Results

### General characteristics of annotated plasmids from non-tuberculous mycobacteria

**Fig. 1:**
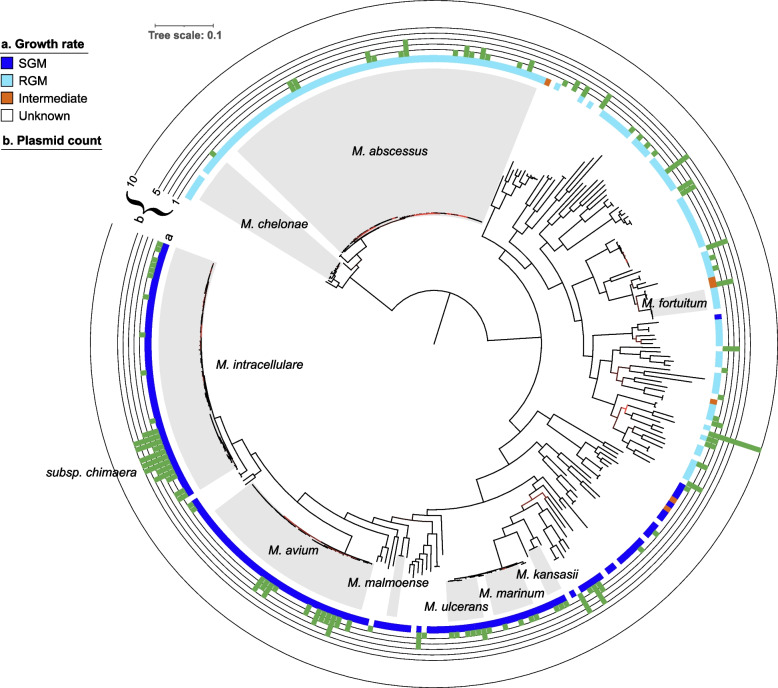
Phylogeny and plasmid content of 328 complete genomes from non-tuberculous mycobacteria. The phylogeny was reconstructed from an alignment of 291 single-copy gene families present on the chromosomes of all strains. The tree scale is in substitutions per site. Branches having a low bootstrap support (< 70%) are colored red. The species most clinically relevant to humans are shaded grey. The number of plasmids in each genome is shown in the form of green bars. SGM: slowly growing mycobacteria, RGM: rapidly growing mycobacteria

To characterize NTM plasmids, we downloaded all publicly available NTM genome assemblies from the NCBI database that were labeled as “complete.” These genomes are expected to be fully assembled, including a (circular) closed chromosomal DNA sequence represented as a single gapless contig, as well as plasmids, if present, which would be represented as additional contigs. In total, 328 unique complete genomes belonging to 125 NTM species were available, including 196 contigs that were annotated in NCBI as plasmids (Additional file 1: Table S1 and S2). The majority of genomes belonged to the most clinically relevant NTM species: *M. abscessus* (*n* = 63), *M. intracellulare* (*n* = 50), and *M. avium* (*n* = 35). Plasmids were present in approximately 30% of the complete genomes (98/328) and 34% (43/125) of NTM species. Out of 98 plasmid-bearing genomes, 49 (50%) contained more than one plasmid, mostly between two and five (Fig. 1 and Additional file 2: Fig. S1). One genome (isolate SMC-4, belonging to a novel NTM species) was annotated with 10 plasmid sequences; however, this likely represents an incompletely assembled plasmidome. Plasmid sequences were present in both human pathogenic and non-pathogenic NTM strains and both rapidly and slowly growing species (Fig. 1).

The plasmid size ranged between 1489 bp (pSMC-4_10) and 864,257 bp (pJCM12687 from *M. branderi*) with a median of 39 kbp. However, the majority (97%) of plasmids had a length between 10 and 400 kbp (Additional file 2: Fig. S1). The number of predicted open reading frames (ORF), i.e., putative protein coding regions, ranged between 1 (pSMC-4_10) and 813 with a median of 32 genes (Additional file 2: Fig. S1). The plasmid GC content varied between 60 and 69% (Additional file 1: Table S2), which is within the range of the chromosomal GC content. Of the 196 plasmids, 34 (17%) were annotated in NCBI as linear plasmids. However, we found inverted terminal repeats (TIR)—similar to NTM plasmid pCLP that was shown to be linear in vitro [54]—only in six of these sequences. On the other hand, for five presumed linear plasmids, the ends of the sequences were nearly identical to their beginnings, indicating potential circularity (Additional file 2: Fig. S2). In contrast to the *M. abscessus* outbreak plasmid pBRA100 that belongs to IncP-1β, none of the 196 other NTM plasmids belonged to a known incompatibility group.

### Cluster analysis and distribution of annotated plasmids from non-tuberculous mycobacteria

**Fig. 2:**
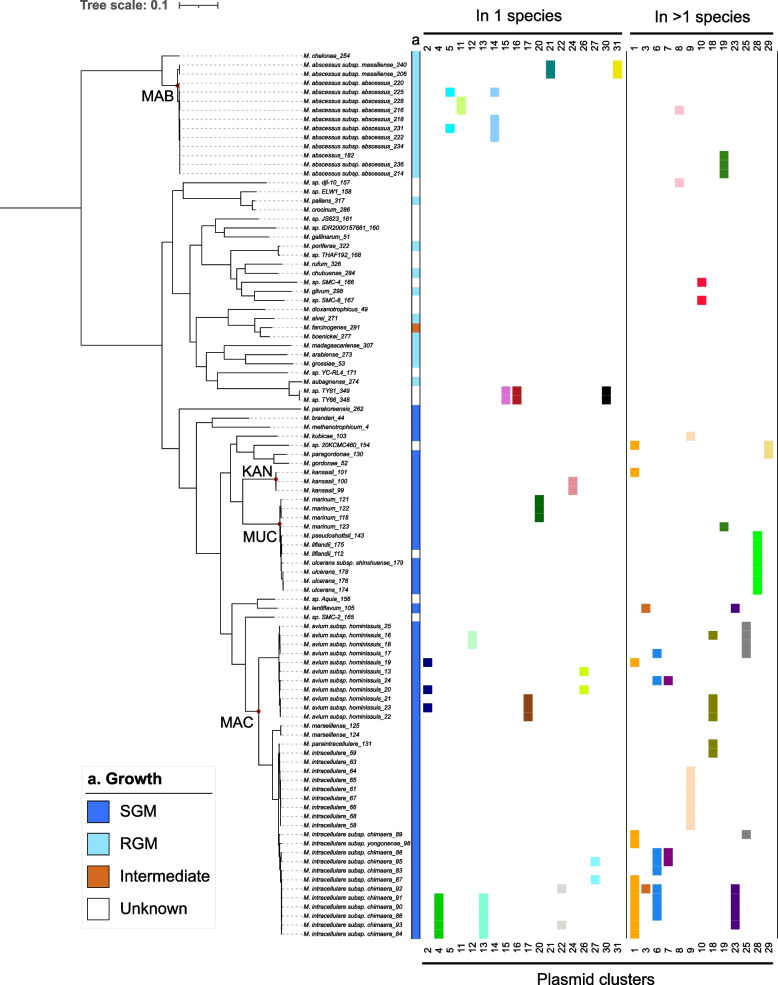
Phylogeny and distribution of plasmid clusters across 98 plasmid-bearing complete genomes from non-tuberculous mycobacteria. The phylogeny was reconstructed from 858 single-copy genes and midpoint rooted. Clinically relevant species are indicated with red dots: MAB = *M. abscessus*, KAN = *M. kansasii*, MUC = *M. ulcerans* complex, MAC = *M. avium* complex. For each plasmid cluster, a different color was used

To identify closely related plasmids, we grouped the 196 annotated plasmids from complete genomes into clusters of homologous sequences using pairwise mash distances. In total, 31 plasmid clusters were identified comprising between 2 and 12 plasmids, leaving 80 plasmids unclustered (Additional file 1: Table S2 and Additional file 2: Fig. S3). Distribution of lengths and similarity values for clustered plasmids are displayed in Additional file 2: Fig. S4. Out of these 31 clusters, 13 comprised plasmids from more than one NTM species (Additional file 1: Table S2 and Fig. 2).

However, seven more clusters and 23 unclustered plasmids (including 6 plasmids from the potentially incomplete SMC-4 genome) were found in more than one NTM species when screening an additional 3755 draft (i.e., incomplete) assemblies for these sequences (Additional file 2: Fig. S5 and Additional file 1: Table S4). Only half of the plasmids that were found in more than one species were annotated as being mobilizable according to the PLSDB metadata information. In total, seven unclustered plasmids (including four from SMC-4) and four plasmid clusters were found both in RGM and SGM species (Additional file 2: Fig. S5 and Additional file 1: Table S4).

**Fig. 3:**
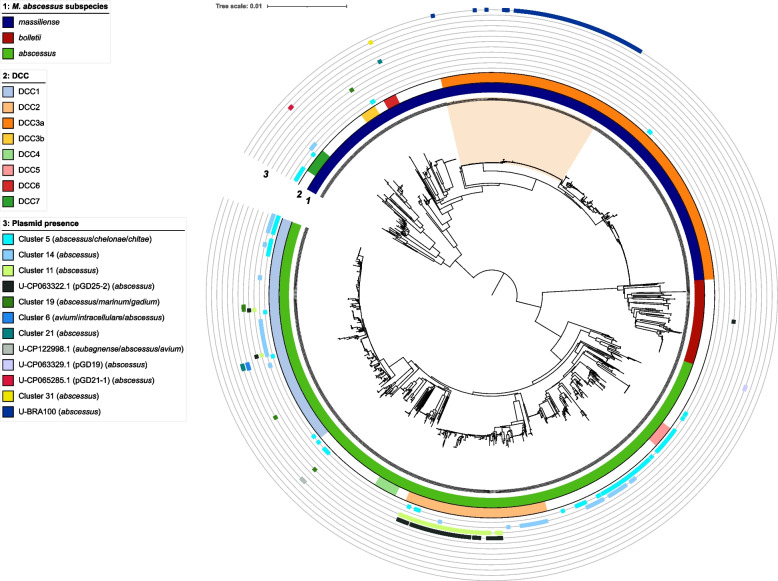
Predicted plasmid distribution in 1486 *M. abscessus* isolates. Illumina short-read sequence data were screened for the presence of 112 annotated plasmid sequences from non-tuberculous mycobacteria. Potential novel plasmids were not predicted. If a plasmid sequence was also identified in draft genomes of species other than *M. abscessus*, those species are indicated in brackets. Hits with annotated plasmid sequences from SMC-4 are not shown. The BRA-100 clade, including isolates belonging to the surgery-related outbreak in Brazil, is shaded light orange. DCC = dominant circulating clone

**Fig. 4:**
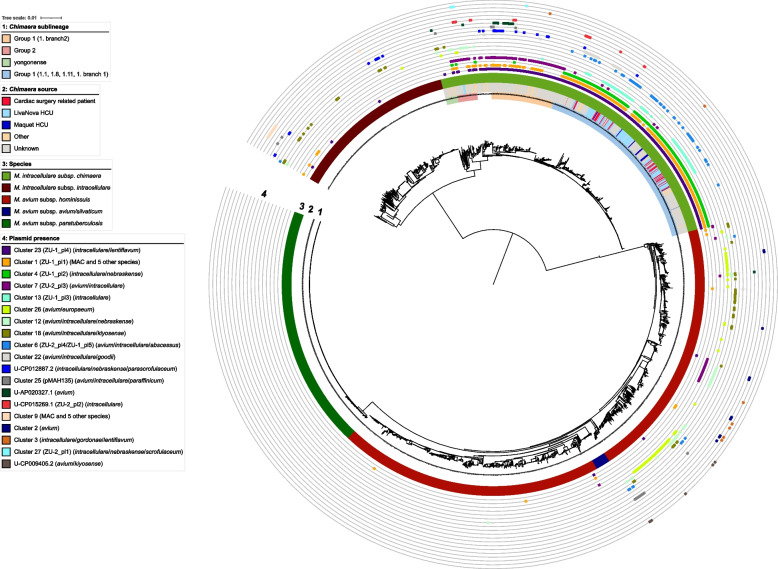
Predicted plasmid distribution in 2451 *M. avium* complex isolates. Illumina short-read sequence data were screened for the presence of 112 annotated plasmid sequences from non-tuberculous mycobacteria. Potential novel plasmids were not predicted. If a plasmid sequence was also identified in draft genomes of species other than *M. avium complex*, those species are indicated in brackets. Only plasmids found in more than six isolates are visualized. Hits with annotated plasmid sequences from SMC-4 are not shown

**Fig. 5:**
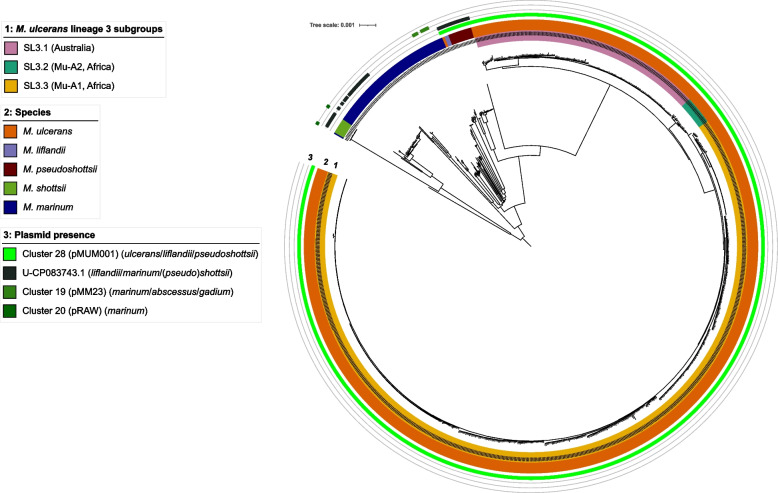
Predicted plasmid distribution in 983 *M. ulcerans* complex isolates. Illumina short-read sequence data were screened for the presence of 112 annotated plasmid sequences from non-tuberculous mycobacteria. Potential novel plasmids were not predicted. If a plasmid sequence was also identified in draft genomes of species other than *M. ulcerans complex*, those species are indicated in brackets

**Fig. 6:**
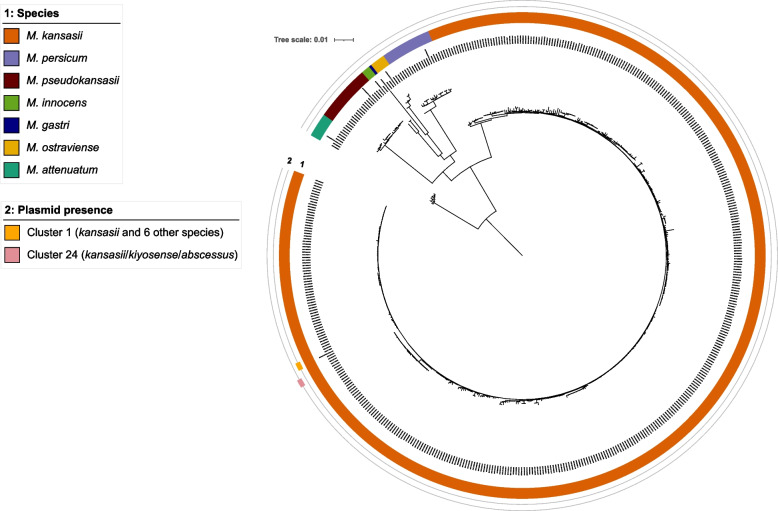
Predicted plasmid distribution of annotated NTM plasmids in 495 *M. kansasii* complex isolates. Illumina short-read sequence data were screened for the presence of 112 annotated plasmid sequences from non-tuberculous mycobacteria. Potential novel plasmids were not predicted. If a plasmid sequence was also identified in draft genomes of species other than *M. kansasii complex*, those species are indicated in brackets

To have a more detailed look at the distribution of putative NTM plasmids across the phylogeny in clinically relevant NTM species, we also searched for the annotated NTM plasmid sequences in short-read sequencing data of 1486 M*. abscessus* isolates, 2451 M*. avium* complex isolates, 983 M*. ulcerans* complex isolates and 495 M*. kansasii* complex isolates (Figs. 3, 4, 5, and 6 and Additional file 1: Table S3).

For *M. abscessus*, plasmid sequences from cluster 5 (pGD42-1, 25 kbp), cluster 14 (pGD42-2, 9.5 kbp), cluster 11 (pGD25-1, 31 kbp) and plasmid pGD25-2 (27 kbp) were most frequently detected in 142 (10%), 129 (9%), 100 (7%), and 87 (6%) strains, respectively (Fig. 3). The former two were found in a more diverse set of isolates while the latter two were mainly confined to some but not all strains belonging to global circulating clone DCC2 (subsp. *abscessus*). Resistance plasmid pBRA100 was only detected in *M. abscessus* strains from the surgery-related outbreak in Brazil.

Within the *M. avium* complex (Fig. 4), several plasmids were detected in multiple MAC species (e.g., clusters 6 and 7). However, the distribution of putative plasmid sequences highly depended on species, subspecies, and subgroups. For example, none of the annotated plasmid sequences from complete NTM genomes were found in *M. avium* subsp. *paratuberculosis* and *M. avium* subsp. *avium* isolates and also one clade of *M. avium* subsp. *hominissuis* seems to be devoid of these sequences. In addition, these plasmid sequences were detected only in certain *M. intracellulare* subsp. *intracellulare* isolates (Fig. 4). On the other hand, nearly all *M. intracellulare* subsp. *chimaera* isolates were predicted to have at least one known plasmid. Within subsp. *chimaera*, additional differential patterns of plasmid presence are observed in different subgroups (e.g., group 1.branch2 and other group 1 isolates). Interestingly, one plasmid from the HCU outbreak clone (ZU-1_pl3) seems to be restricted mainly to isolates from cardiac surgery-related patients and heater-cooler units (Fig. 4).

With regard to *M. ulcerans* complex (Fig. 5), plasmid sequences from cluster 28 (including the notorious virulence plasmid pMUM001) were found in all but one *M. ulcerans* strain (*n* = 858), all 17 M*. pseudoshottsi* and all three *M. liflandii* strains (the latter two regarded as ecovars of *M. ulcerans*) and missing in all *M. marinum* and *M. shottsii* strains. In addition, plasmid CP083743.1 was found in all but one *M. shottsii*, one group of *M. marinum* strains, and in *M. pseudoshottsi* and *M. liflandii* but not lineage 3 M*. ulcerans* (Fig. 5).

Within 495 M*. kansasii* complex isolates (Fig. 6), known plasmid sequences were rarely found with only 2 plasmid clusters (1 and 24) identified in 4 isolates.

To further analyze the extent of putative inter-species plasmid transfer, we also compared the set of 196 annotated NTM plasmids from complete genomes with 59, 687 plasmids from other bacteria included in the curated plasmid database PLSDB [41] (Additional file 1: Table S5). The only NTM plasmid that was closely related to a non-mycobacterial plasmid was CP079876.1, a 12-kbp plasmid from *Mycobacterium* sp. SMC-4, showing > 99% ANI and 100% alignment fraction with *Rhodococcus* sp. plasmids that were at least 79 kbp (Additional file 1: Table S6).

### Plasmid diversity within a single non-tuberculous mycobacteria strain

Next, we focused on intra-strain plasmid diversity, comparing plasmids present within individual strains. Of the 49 complete genomes with multiple plasmids, 14 harbored exclusively small (< 50 kbp, 29%), 10 only large (> 50 kbp, 20%), and 25 (51%) genomes carried both types of plasmids (Additional file 2: Fig. S6). Plasmids within the same strain generally exhibited low sequence similarity, reflected by high pairwise mash distances and the classification into different plasmid clusters (Additional file 2: Fig. S6). However, one *M. avium* strain harbored two plasmids (NZ_CP040251.1 and NZ_CP040252.1) that were very closely related and belonged to the same plasmid cluster (Additional file 2: Fig. S6, organism ID 23 and Additional file 2: Fig. S7). Detailed analysis of this pair of plasmids revealed that, despite their high similarity (ANI > 99%), they differed significantly because each plasmid contained a different region that was duplicated (Additional file 2: Fig. S7–S9). The duplicated regions (9393 and 9049 bp) within NZ_CP040251.1 displayed 92% identity with each other with several smaller and larger indels (ranging from 1 to 141 bp) and contained the insertion element IS21 with transposases (istA/B) as well as recombinases (xerC_1/2) (Additional file 2: Fig. S8). The duplicated regions in NZ_CP040252.1 (9080 and 9111 bp) displayed 98% identity with each other with only smaller indels (ranging from 1 to 6 bp) and contained other transposases and recombinases (recD2_1/2) (Additional file 2: Fig. S9).

### The protein repertoire of annotated plasmids from non-tuberculous mycobacteria

To study the protein repertoire of NTM plasmids in more detail, we first clustered all genes in the analyzed genomes into protein families based on amino-acids sequence similarity. The clustering yielded a total of 36,180 protein families, of which 1041 families included only plasmid-borne genes (i.e., these families are plasmid-specific). Additionally, 3902 protein families included member genes found on both plasmids and chromosomes. A total of 1686 plasmid genes could not be clustered into a protein family (i.e., they remained as singletons). The size of plasmid protein families ranged between 1 and 98, with a median of 2 genes per protein family (Additional file 1: Table S7). Hence, a large portion of the genes are shared only by a limited number of plasmids and no protein family was universally found encoded on all annotated NTM plasmids (Additional file 1: Table S8 and Additional file 2: Fig. S10).

Out of 17,547 protein-coding genes found on the 196 plasmids, 8168 (46.5%) were annotated as hypothetical proteins (Additional file 1: Table S7). The most prevalent proteins with functional annotation were replication initiator proteins (e.g., repA), ATP binding proteins, plasmid-segregation related proteins (e.g., parA), recombinases/integrases, components of the type VII secretion system (e.g., WXG100 and PPE), transposases, transcriptional regulators, hydrolases and mobility-related proteins (e.g., MobF relaxases) (Additional file 1: Table S7 and Table S8). Additionally, a significant number of genes were annotated as putative toxin/antitoxin system (*n* = 360) components and IS transposases (*n* = 398) belonging to 26 different IS families (Additional file 1: Table S7). Proteins with the same predicted function (e.g., repA and mobF) typically belonged to several protein families (Additional file 1: Table S8). Notably, prediction of mobility, conjugation, and replication genes greatly differed between different plasmid prediction tools (Additional file 1: Table S2).

**Fig. 7:**
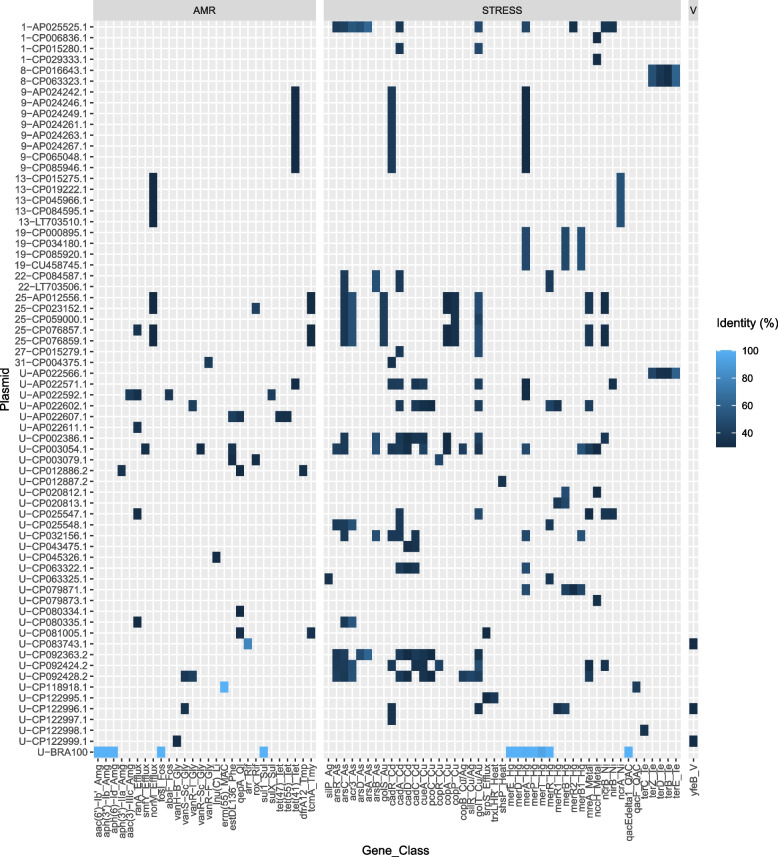
Heatmap of putative resistance, stress, and virulence genes found in 197 annotated plasmid sequences from non-tuberculous mycobacteria using AMRfinder + . Only genes with no internal stopcodons, > 30% amino acid identity and > 70% coverage compared to markers in the AMRfinder + reference database are shown. Plasmid accession numbers are preceded by the plasmid cluster number. U = unclustered, AMR = antimicrobial resistance, V = virulence. Sul = sulfonamide, Amg = aminoglycoside, MAC = macrolide, Rif = rifamycin, Tmy = Tetracenomycin, Phe = phenicol, Ql = quinolone, Li = lincosamide, Tmp = trimethoprim, As = arsenic, Cd = cadmium, Cu = copper, Au = gold, Ag = silver, Hg = mercury, Ni = nickel, QAC = quaternary ammonium compound, Te = tellurium, Tet = tetracycline, Fos = Fosfomycin, Gly = glycopeptide

With the default settings of AMRfinder + , no stress resistance or virulence genes were detected on the 196 annotated NTM plasmids from complete genomes, and only one putative antimicrobial resistance gene was identified: the methyltransferase erm(55) encoded on the *M. chelonae* pMchErm55 plasmid, which may confer inducible resistance to macrolides (Additional file 1: Table S9 and Fig. 7). In addition, 5 putative resistance genes were found on the BRA100 plasmid as expected (Additional file 1: Table S9 and Fig. 7). Using more relaxed detection parameters (> 30% amino acid identity and > 70% coverage), we found putative homologs without internal stop codons of known antimicrobial resistance (AMR) proteins in 36 out of 196 (18%) plasmids and of stress resistance proteins (e.g., metal resistance) in 60 out of 196 (31%) plasmids (Fig. 7 and Additional file 1: Table S9). The identified homologs might confer resistance against a wide range of metals and antibiotics including tetracycline, fosfomycin, sulfonamide, aminoglycoside, phenicol, quinolones, trimethoprim, lincosamide, macrolides, and rifamycin. Two putative aminoglycoside resistance genes, *aph(3')-IIa*) and *aac(3)-IIIc,* were found on a plasmid from *M. intracellulare* and *M. arabiense*, respectively. The putative rifamycin resistance gene *rox* was detected on a plasmid from another *M. intracellulare* strain and on a plasmid from an unknown species. No homologs of beta-lactamases were detected on the NTM plasmids.

Polyketide synthase genes responsible for the mycolactone toxin causing Buruli ulcers were only found in *M. ulcerans* complex isolates (Additional file 1: Table S10). Nocobactin polyketide synthase NbtC (mycobactin), found on the virulence plasmid pMAH135 (AP012556), was also found in all other plasmids belonging to the pMAH135 plasmid cluster as well as in another unclustered plasmid from *M. intracellulare* (CP023152.1) (Additional file 1: Table S7). However, none of these polyketide synthase genes were detected by AMRfinder + . The only putative virulence gene that was detected with AMRfinder + using relaxed thresholds was *yfeB*, which showed only low amino acid identity (31%) to known reference proteins (Fig. 7 and Additional file 1: Table S9). Homologs of putative virulence genes included in the virulence factor database [47] were found in 47 plasmids (24%) including polyketide synthases (pks15), phospholipases (plcA/B/C), fibronectin-binding proteins (fbpA/D), and ESX components (eccA5/B5/C5, esxG/H/M/N, mycP1/P3/P5, PE5/18/19) (Additional file 1: Table S10).

## Discussion

In this study, we demonstrate that plasmids are prevalent across both slowly and rapidly growing NTM species, including human pathogens and clinically irrelevant strains. However, the presence of these plasmids is inconsistent across species, subspecies, and even within (sub)lineages, with some strains carrying a specific plasmid while others do not. NTM plasmids are highly diverse with many uncharacterized genes and only a limited number of known resistance or virulence-associated genes. Closely related plasmids were frequently found in different NTM species, suggesting that plasmid-mediated horizontal gene transfer may play an important role in NTM evolution.

Sequences annotated as plasmids were present in about 30% of all NTM species for which complete genomes were available in NCBI, including clinically relevant species such as *M. abscessus*, *M. avium*, *M. intracellulare*, *M. kansasii*, *M. marinum*, and *M. ulcerans*. However, with only a limited number of complete genomes currently available for many NTM species, more plasmids are expected to be discovered as additional sequencing data becomes available. The majority of NTM genomes containing multiple plasmids harbored between two and five plasmids. The genome of *Mycobacterium* sp. SMC-4, however, included a closed circular chromosomal sequence and an unusually high number of 10 sequences annotated as plasmids (eight of which were linear and three ≤ 5 kbp) suggesting these contigs may not represent fully assembled functional plasmid entities.

Within NCBI, actually, a large number of NTM plasmids are labeled as linear (17%). Linear NTM plasmids with invertron-like structures (i.e., with terminal inverted repeats) and lengths between 15 and 320 kbp have been described for several NTM including *M. xenopi*, *M. branderi*, *M. intracellulare*, *M. celatum, M. abscessus*, and *M. avium* [54–59]. Their topology was confirmed by PFGE migration patterns, sensitivity to exonuclease III (which degrades DNA from free 3′ ends), sensitivity to exonuclease lambda (which degrades DNA from free 5′ ends), topoisomerase (which relaxes circular plasmids, changing migration speed) insensitivity and/or RFLP analysis. TIRs were not identified in the SMC-4 sequences nor in most other supposed linear plasmid sequences; however, for some, the ends of the sequences closely matched their beginnings, though not perfectly, suggesting they may be circular but were likely affected by assembly challenges. In addition, we also observed several inconsistencies in submitted annotations. For example, pMyong2, a plasmid from *M. intracellulare* was experimentally verified to be linear [60], but labeled in NCBI as circular. On the other hand, pMUM002 from *M. liflandii* [61] was identified as circular by sequencing of overlapping BAC clones while it is labeled as linear in NCBI. In addition, 10 out of 31 clusters of closely related plasmids, comprised plasmids with different topologies (e.g., cluster 28, comprising pMUM001 plasmid) and different plasmid lengths further indicating potential mislabeling or assembly challenges.

Interestingly, not all strains belonging to the same phylogenetic group harbored the same number or type of plasmids indicating several independent events of plasmid acquisition and loss. Notably, nearly all *M. intracellulare* subsp. *chimaera* isolates harbored multiple plasmids but even within this subspecies, different plasmid presence patterns were observed in different sublineages. In addition, known plasmids were absent in the genomes of *M. avium* subsp. *avium*, the etiological agent of avian tuberculosis*,* and *M. avium* subsp. *paratuberculosis*, a globally important obligate pathogen of domestic and wild ruminants and the causative agent of Johne’s disease [30, 62], while *M. avium* subsp. *hominissuis*, which is typically isolated from humans [30], harbored between 0 and 4 plasmids, potentially reflecting differences in their ecology, pathogenicity, host specificity, plasmid uptake potential, or adaptation strategies.

The absence of a protein family universally present across all annotated NTM plasmids suggests either significant diversity in plasmid backbones, potential inaccuracies in sequence annotation, or a combination of both. Indeed, some sequences labeled as plasmids in NCBI may in fact represent misannotated chromosomal fragments, genomic islands, or other mobile genetic elements. However, the in silico validation of the analyzed sequences as true plasmids remains difficult for two reasons. First, many in silico plasmid prediction tools rely on, or are trained with, plasmid data derived from NCBI, i.e., the same data used in this study. Any incorrectly annotated sequence in NCBI can therefore also introduce biases into these prediction tools, resulting in the classification of non-plasmid sequences as plasmids. On the other hand, current plasmid prediction tools may fail to identify true plasmid sequences due to reliance on outdated databases, overly strict filtering criteria (e.g., plasmid length thresholds unsuitable for NTM plasmids), or an inability to detect highly divergent plasmid backbone genes (e.g., replication genes). This might be particularly problematic for NTM plasmids, as there is a lack of experimental verification to confirm their existence, replication mechanisms, and other key features.

Genes most prevalent on the presumed NTM plasmids were either annotated as hypothetical proteins or mostly related to basic plasmid functions such as replication (e.g., repA), maintenance of plasmid copy number and evolution (e.g., recombinases/integrases), segregation (e.g., toxin/anti-toxin systems), mobilization (e.g., mob relaxases) and conjugation (e.g., type VII secretion system). Putative resistance genes were identified in both human-pathogenic and non-pathogenic mycobacterial plasmids, though only a few are predicted to confer resistance to the antibiotics most commonly used to treat NTM infections: aminoglycosides, macrolides, and rifamycin. Additionally, amino acid identity compared to resistance proteins in the AMRfinder + reference database was typically low (< 55%), underscoring the need for in vitro (e.g., phenotypic drug susceptibility testing) and in vivo (e.g., using antibiotic-treated infected mice) experiments to confirm their function in NTM. This is further highlighted by the fact that the notorious IncP1 multi-drug plasmid (NC_017908.2, BRA100) from the *M. abscessus* subsp. *massiliense* strain that caused the nation-wide post-surgical infection outbreak in Brasil [9, 10] contains three genes that encode putative resistance against aminoglycoside antibiotics. However, all investigated epidemic Brazilian strains so far showed phenotypic susceptibility against amikacin [29–32]. Genes for known beta-lactamases were solely found on the chromosomes and not on NTM plasmids. The *erm(55)*^*P*^ gene, recently identified as potentially conferring plasmid-mediated inducible macrolide resistance in *M. chelonae*, was found in our dataset solely on the original plasmid pMchErm55 [63, 64] and in one *M. obuense* draft genome. Nonetheless, continued surveillance of both the gene and plasmid is advised.

The only putative virulence sequence that was detected with AMRfinder + relaxed parameter settings was *yfeB*, found on one plasmid belonging to the pathogen *M. ulcerans* and on two plasmids from *M. aubagnense*, which rarely causes disease in humans. This gene, coding for an iron/manganese ABC transporter ATP-binding protein, was described in the plague pathogen *Yersinia pestis*, where it was shown to play an important role in iron acquisition and virulence [65]. However, given that the putative *yfeB* sequences encoded on the NTM plasmids share only 31% amino acid identity with those from *Yersinia*, there is a fair chance that these proteins may not perform the same role. Additional homologs of *M. tuberculosis* virulence factors [47] that were not detected by AMRfinder + were found in almost 25% of NTM plasmids but at least some of them might also be pseudogenes. Plasmids closely related to the supposed virulence plasmid pMAH135 [13] were only found in members of the MAC.

Clustering or classifying plasmids is crucial for understanding their genetic diversity, evolutionary relationships, and functional roles, as well as for tracking the spread of antibiotic resistance and virulence factors. Until now, plasmid classification and characterization efforts have mainly been focused on plasmids from *Enterobacteriaceae* [40, 66]*.* Based on sequence similarity of their replication genes and the inability to coexist in the same cell, *Enterobacteriaceae* plasmids have been classified into so-called incompatibility (inc) groups [67]. In addition, inc groups can be subtyped using plasmid multi-locus sequence typing (pMLST) [40], and transmissible plasmids can be classified based on relaxase genes (MOB-typing). Identified mobility proteins from NTM plasmids all belonged to two out of six known MOB families [68], i.e., mobF and mobP. However, only the plasmid from the *M. abscessus* clone BRA-100 could be assigned to an existing incompatibility group, demonstrating that this typing method has limited applicability for NTM.

Therefore, we clustered the annotated NTM plasmids based on their overall sequence similarity to one another and screened for their presence in draft assemblies and short-read sequencing data from thousands of NTM isolates. We observed that many closely related plasmids were shared among multiple NTM species, including some distantly related species, while others were restricted to specific phylogenetic groups within a species. This suggests that both horizontal gene transfer and vertical inheritance are likely mechanisms of plasmid acquisition within NTM. Horizontal gene transfer across species might be facilitated by the fact that different NTM species can occupy the same environmental niche [69, 70] and can also co-colonize or infect patients simultaneously [25, 71–73]. On the other hand, some detections of plasmids in NTM species other than the original host may also be due to low-level undetected contamination, potentially from cultures that were not thoroughly subcultured to achieve pure isolates.

A limitation of this study is its reliance solely on in silico data. Although all 196 analyzed putative plasmid sequences were derived from complete genomes, labeled as plasmids in the NCBI database, and included in the curated plasmid database PLSDB [41], we cannot rule out the possibility that some sequences, apart from those of SMC-4, may be incomplete or incorrectly classified as plasmids. In addition, we did not specifically search for novel plasmids, as reconstructing complete plasmid sequences from short-read data remains extremely challenging [74], particularly for NTM genomes, which often harbor multiple large plasmids. Still, even long-read sequencing techniques like PacBio and Nanopore, which are typically well-suited for fully assembling plasmid sequences, seem to struggle with this, at least for some NTM isolates. Additionally, it must also be noted that plasmids may have been lost during subculturing, DNA extraction, or library preparation prior to sequencing biasing prevalence numbers. Lastly, as with plasmid backbone genes, it is also possible that putative resistance and virulence genes located on NTM plasmids are overlooked, i.e., not detected with current in silico prediction tools, because they are not yet well-characterized or not included in current reference databases. To address this, we also applied more relaxed detection thresholds, though this comes with the drawback of potentially increasing false positive results [45].

## Conclusions

This study highlights the widespread presence and remarkable diversity of putative plasmids in non-tuberculous mycobacteria, with significant variation in their distribution across species, subspecies, and even (sub)lineages. The findings also suggest that both horizontal gene transfer and vertical inheritance contribute to plasmid evolution in NTM. Challenges in plasmid assembly, annotation accuracy, and gene function prediction underscore the need for further experimental validation and additional sequencing efforts to better understand the functional roles of NTM plasmids.

## Supplementary Information

Additional file 1. Supplementary Tables. This file contains all Supplementary Tables and their corresponding legends.

Additional file 2. Supplementary Figures. This file contains all Supplementary Figures and their corresponding legends.

## Data Availability

Accession numbers of all genomes and plasmids used in this study are available in Additional file 1: Table S1 and S2. The corresponding sequence data is publicly available at NCBI (https://www.ncbi.nlm.nih.gov/). All software tools used in this study are open-source and custom scripts are available at github [52, 75].

## Abbreviations

AMR: Antimicrobial resistance
DCC: Dominant circulating clone
INC: Incompatibility
pMLST: Plasmid multi-locus sequence typing
MAC: *Mycobacterium avium* Complex
MCL: Markov Cluster Algorithm
NCBI: National Center for Biotechnology Information
NTM: Non-tuberculous mycobacteria
ORF: Open reading frame
RBH: Reciprocal best hits
TIR: Terminal inverted repeat
VFDB: Virulence factor database
