# Supplementary material for: Genomic insights into the plasmidome of non-tuberculous mycobacteria
Source: Genome Med. 2025 Mar 4;17:19. doi: 10.1186/s13073-025-01443-7 (PMC11877719; doi:10.1186/s13073-025-01443-7)
Supplement: Supplementary file 1 — Additional file 1. Supplementary Tables. This file contains all Supplementary Tables and their corresponding legends. [file 13073_2025_1443_MOESM1_ESM.zip › Additional file 1/Supplementary_Tables_legends.docx]

**Supplementary Table Legends for**

**Genomic insights into the plasmidome of non-tuberculous mycobacteria**

Margo Diricks*^1,2,3^, Florian P. Maurer^3,4^, Viola Dreyer^1,2,3^, Ivan Barilar^1,2,3^, Christian Utpatel^1,2,3^, Matthias Merker^2,5^, Nils Wetzstein**^†^**^1,6^, Stefan Niemann**^†^**^1,2,3^

^1^Molecular and Experimental Mycobacteriology, Research Center Borstel, Borstel, Germany

^2^German Center for Infection Research (DZIF), partner site Hamburg-Lübeck-Borstel-Riems, Borstel, Germany

^3^National and WHO Supranational Reference Laboratory for Mycobacteria, Research Center Borstel, Leibniz Lung Center, Borstel, Germany

^4^Institute of Medical Microbiology, Virology and Hygiene, University Medical Center Hamburg-Eppendorf, Hamburg, Germany

^5^Evolution of the Resistome, Research Center Borstel, Borstel, Germany

^6^Goethe University Frankfurt, University Hospital, Department of Internal Medicine, Infectious Diseases, Frankfurt am Main, Germany.

**†**These authors contributed equally

*Corresponding author: Dr. ir. Margo Diricks (mdiricks@fz-borstel.de)

Keywords:

Non-tuberculous mycobacteria, plasmids, genomics, antimicrobial resistance

**Table S1:** Overview of all complete and draft genomes from non-tuberculous mycobacteria used in this study.

**Table S2:** Overview of all contigs from complete genomes of non-tuberculous mycobacteria used in this study.

**Table S3:** Overview of all short-read sequencing datasets from non-tuberculous mycobacteria used in this study.

**Table S4:** Results of fastANI analysis between NTM plasmids and draft genomes from non-tuberculous mycobacteria.

**Table S5:** Overview of all plasmids included in the PLSDB database v2023-11-03_v2.

**Table S6:** Results of fastANI analysis between plasmids from PLSDB database and 196 annotated plasmids from non-tuberculous mycobacteria.

**Table S7:** Overview of protein-coding genes found on 196 annotated plasmids from non-tuberculous mycobacteria.

**Table S8:** Top 20 most prevalent proteins encoded on 196 annotated plasmids from non-tuberculous mycobacteria. *^1^Total number of genes annotated with this protein name. Protein names were extracted from the Refseq annotation files. ^2^Total number of plasmids that contained at least one gene annotated with this protein name. ^3^Total number of protein families that contained at least one gene annotated with this protein name. Note that within the same protein family, genes can have a different protein name.*

**Table S9:** Putative resistance, stress and virulence genes identified on 196 annotated plasmids from non-tuberculous mycobacteria using AMRfinder+ with relaxed thresholds of 30% amino acid identity and 70% coverage

**Table S10:** Putative virulence genes found on 196 annotated plasmids from non-tuberculous mycobacteria using abricate and the virulence factor reference database.
