# Supplementary material for: Genomic insights into the plasmidome of non-tuberculous mycobacteria
Source: Genome Med. 2025 Mar 4;17:19. doi: 10.1186/s13073-025-01443-7 (PMC11877719; doi:10.1186/s13073-025-01443-7)
Supplement: Supplementary file 2 — Additional file 2. Supplementary Figures. This file contains all Supplementary Figures and their corresponding legends. [file 13073_2025_1443_MOESM2_ESM.zip › Additional file 2/Supplementary_Figures_legends.docx]

**Supplementary Figure Legends for**

**Genomic insights into the plasmidome of non-tuberculous mycobacteria**

Margo Diricks*^1,2,3^, Florian P. Maurer^3,4^, Viola Dreyer^1,2,3^, Ivan Barilar^1,2,3^, Christian Utpatel^1,2,3^, Matthias Merker^2,5^, Nils Wetzstein**^†^**^1,6^, Stefan Niemann**^†^**^1,2,3^

^1^Molecular and Experimental Mycobacteriology, Research Center Borstel, Borstel, Germany

^2^German Center for Infection Research (DZIF), partner site Hamburg-Lübeck-Borstel-Riems, Borstel, Germany

^3^National and WHO Supranational Reference Laboratory for Mycobacteria, Research Center Borstel, Leibniz Lung Center, Borstel, Germany

^4^Institute of Medical Microbiology, Virology and Hygiene, University Medical Center Hamburg-Eppendorf, Hamburg, Germany

^5^Evolution of the Resistome, Research Center Borstel, Borstel, Germany

^6^Goethe University Frankfurt, University Hospital, Department of Internal Medicine, Infectious Diseases, Frankfurt am Main, Germany.

**†**These authors contributed equally

*Corresponding author: Dr. ir. Margo Diricks (mdiricks@fz-borstel.de)

Keywords:

Non-tuberculous mycobacteria, plasmids, genomics, antimicrobial resistance

**Fig. S1: Basic characteristics of plasmids from non-tuberculous mycobacteria (NTM).**

(A) Number of plasmids per NTM genome. The final dataset comprised 98 plasmid-carrying genomes with a total of 196 plasmids and 230 plasmid free genomes (not shown) (B) Distribution of plasmid sizes, expressed as number of ORFs per plasmid contig. (C) Distribution of plasmids sizes, expressed as number of base pairs. Note that the unusual high number of 10 plasmids for isolate SMC-4 is likely a result from an assembly artefact.

**Fig. S2: Self-dot plots of plasmids from non-tuberculous mycobacteria annotated as linear in NCBI.**

Dots represent stretches of homology between both sequences. AF312688.1 = pCLP: linearity was proven with nuclease treatment and electrophoretic mobility (Picardeau 1998) in vitro and described to have inverted terminal repeats (ITR). Color of the dot plot according to the length of the match, from blue for short matches, to red for matches over 100 bp long. Only plasmids with special patterns (TIR and other repeats) are visualized. *Self dot plots for AP022611.1 and AP022622.1 looked similar. Dot plots were generated with Geneious using a tile size of 50,000 and word size of 20. TIRs can be found as / lines starting from the right upper corner while sequences of which the end and beginning match (indicating potential circularity) can be identified as \ lines in the right upper corner.

**Fig. S3: Mashtree of 196 annotated plasmids from complete genomes of non-tuberculous mycobacteria.**

The mash-distance based tree was generated using Mashtree^7^. Indicated are the plasmid clusters determined based on mash distances using a threshold of 0.05. Plasmid lengths (bp) are indicated as bars.

**Fig. S4: Characteristics of plasmid clusters from non-tuberculous mycobacteria.**

Plasmid clusters were determined with a mash distance threshold of 0.05. (A) Combined box and jitter plot of plasmid size distribution within plasmid clusters (1-31), U = plasmid sizes of unclustered plasmids. Plasmids having a length above 50 kbp are considered large plasmids. (B) Boxplot of pairwise mash distances between plasmids belonging to the same cluster (1-31), O = pairwise mash distances between unclustered plasmids and clustered plasmids, between plasmids belonging to different clusters and between unclustered plasmids. (C) Boxplot of average nucleotide identity (ANI) between plasmids belonging to the same cluster. (D) Boxplot of alignment fraction between plasmids belonging to the same cluster. Plasmid clusters found in more than one species with complete genome are colored blue. Solid lines indicate median values, box represent interquartile range (IQR), whiskers extend to 1.5 of the IQR, dots represent outliers.

**Fig. S5: Annotated plasmids found in more than one species of non-tuberculous mycobacteria.**

3,755 NTM draft (i.e. incomplete) assemblies belonging to >200 NTM species were screened for 112 annotated NTM plasmids (1 per cluster) using FastANI with a threshold of >95% ANI and >90% alignment fraction. Plasmids found only in multiple novel species (i.e. cluster 10) are not displayed. Panel A: Predicted absence/presence patterns. Novel species are not included. Panel B: Predicted number of species with annotated plasmids. Novel species are not included. SGM = slowly growing mycobacteria, RGM = rapidly growing mycobacteria; * indicate plasmids that are found both in RGM and SGM species, including novel species. Plasmids from the potentially incomplete genome SMC-4 are indicated in red.

**Fig. S6: Characteristics of plasmids from 49 NTM strains harboring more than one plasmid.**

(A) Combined box and jitter plot of lengths of plasmids belonging to the same strain. Plasmid clusters were determined with a mash distance threshold of 0.05. Plasmids having a length above 50 kbp are considered large plasmids. (B) Boxplot of pairwise mash distances between plasmids belonging to the same strain. Solid lines indicate median values, box represent interquartile range (IQR), whiskers extend to 1.5 of the IQR, dots represent outliers.

**Fig. S7: Dot plots comparing sequences of two closely related plasmids (NZ_CP040251.1. and NZ_CP040252.1) from *M. avium* strain 101115 (organism ID 23).**

Dots represent stretches of homology between both sequences. Duplicated regions are indicated with arrows. Dot plots were generated with Geneious using a tile size of 50,000 and word size of 20.

**Fig. S8: Plasmid map of *M. avium* plasmid NZ_CP040251.1.**

Figure created using proksee. Locustags from Genbank file are indicated. Prokka annotation was added between brackets for annotations other than hypothetical proteins.

**Fig. S9: Plasmid map of *M. avium* plasmid NZ_CP040252.1.**

Figure created using proksee. Locustags from Genbank file are indicated. Prokka annotation was added between brackets for annotations other than hypothetical proteins.

**Fig. S10: Dot plot showing the occurrence of clustered proteins on chromosomes and plasmids to show the gene content shared between replicons.**

The colors highlight protein families containing genes commonly associated with plasmid backbones.
