# Supplementary figures and images for: Genomic insights into the plasmidome of non-tuberculous mycobacteria
Source: Genome Med. 2025 Mar 4;17:19. doi: 10.1186/s13073-025-01443-7 (PMC11877719; doi:10.1186/s13073-025-01443-7)

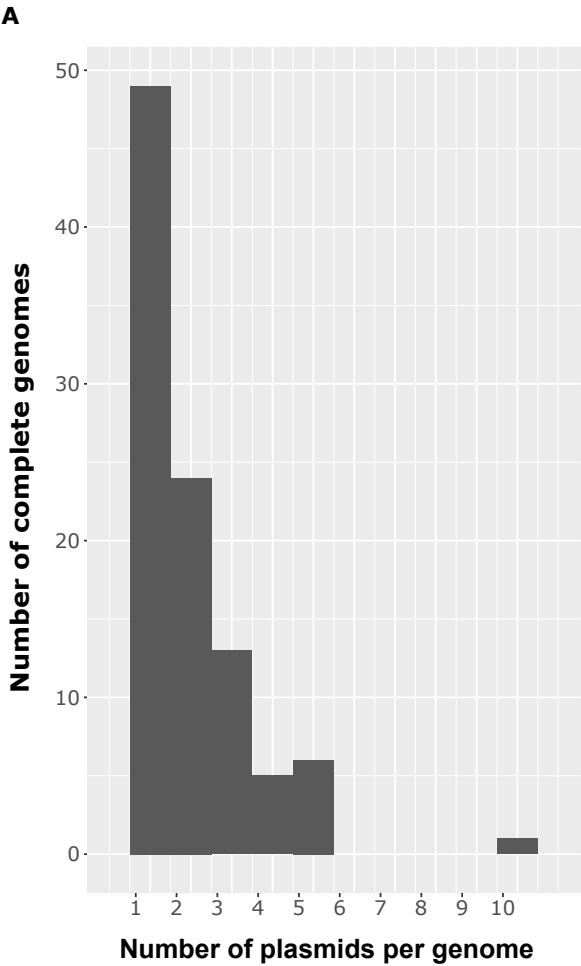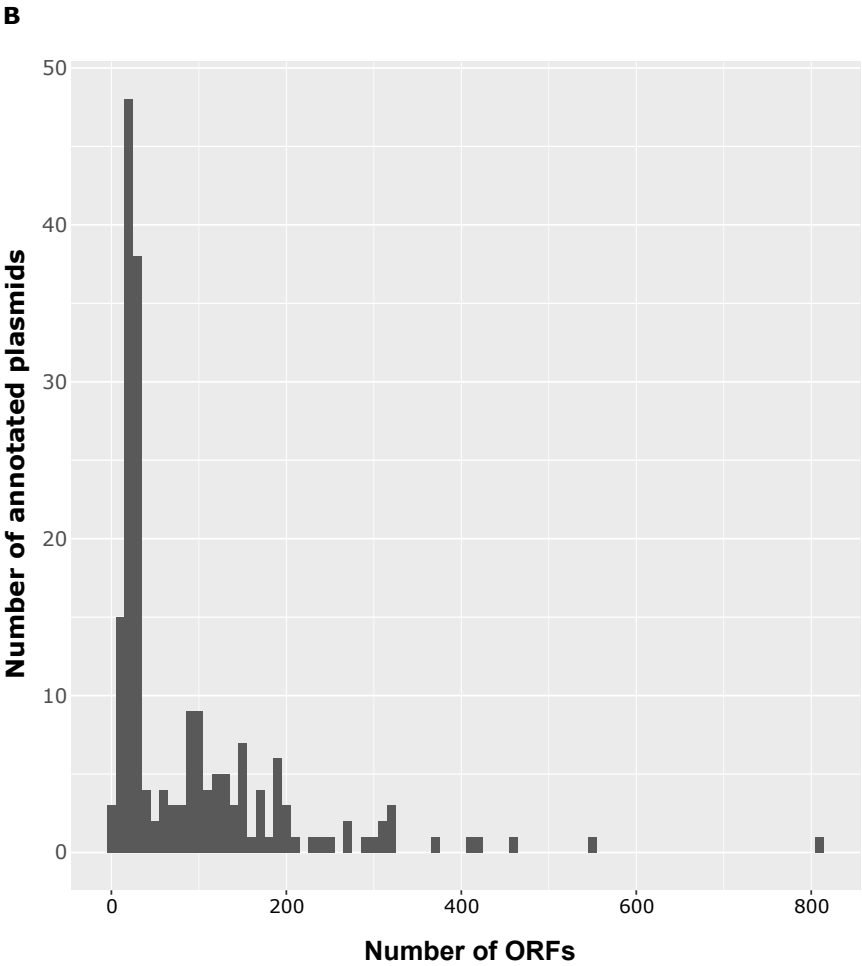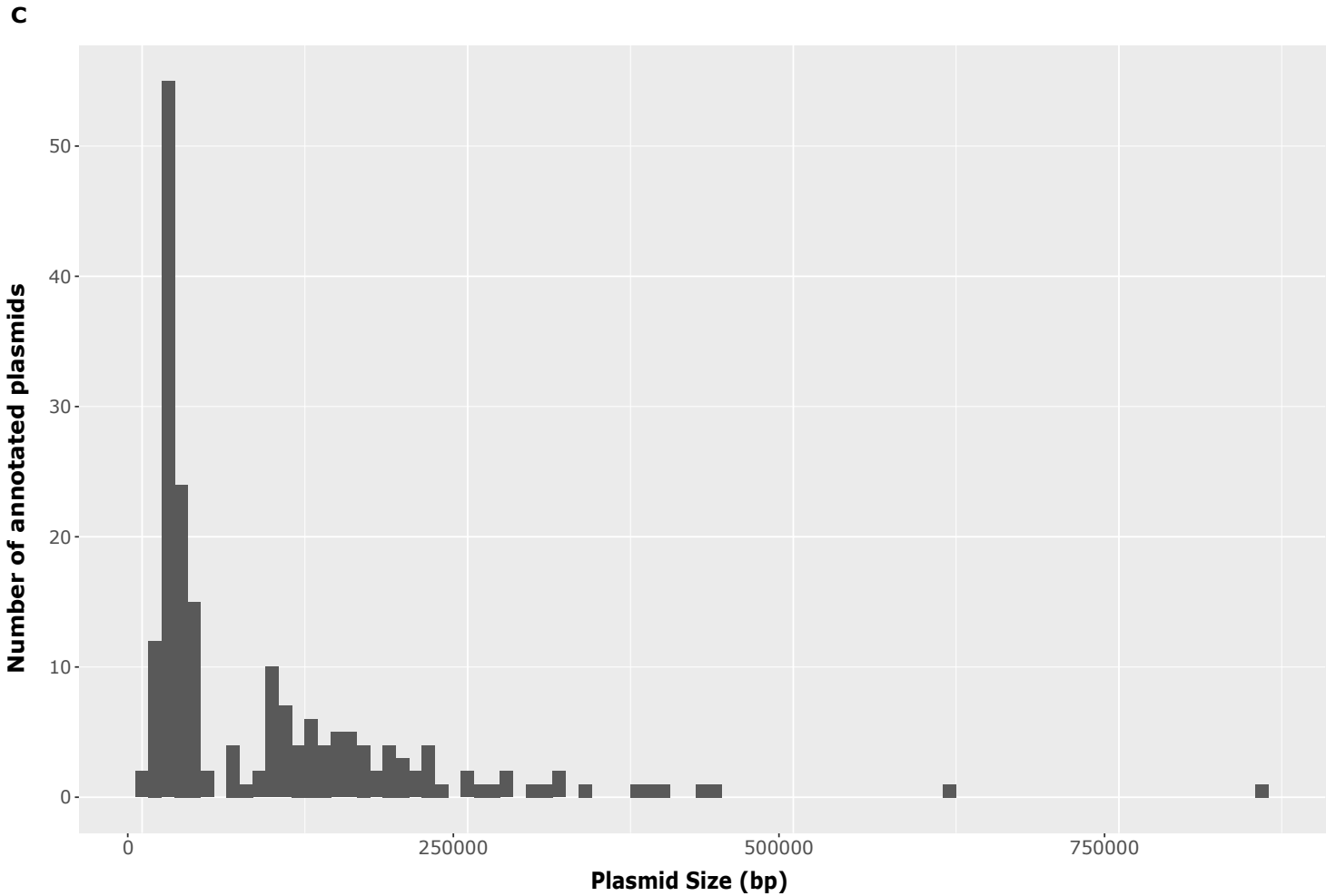

Supplement: Supplementary file 2 — Additional file 2. Supplementary Figures. This file contains all Supplementary Figures and their corresponding legends. [file 13073_2025_1443_MOESM2_ESM.zip › Additional file 2/Fig S1_Characteristics_plasmids.pdf]

Plasmids vs Host Chromosomes Occurance of Protein Clusters

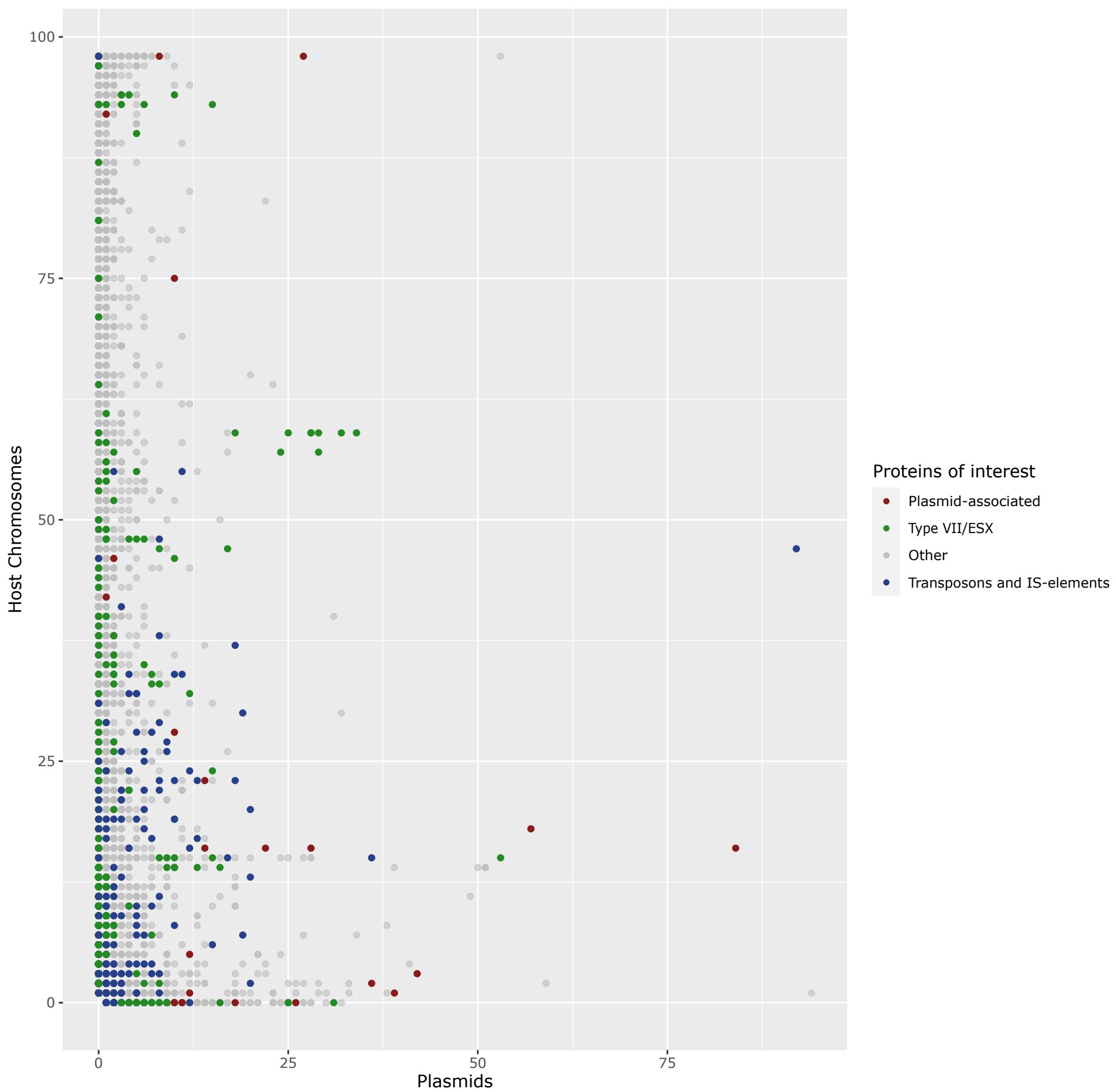

Supplement: Supplementary file 2 — Additional file 2. Supplementary Figures. This file contains all Supplementary Figures and their corresponding legends. [file 13073_2025_1443_MOESM2_ESM.zip › Additional file 2/Fig S10_DotPlot_plasmids_chromosomes.pdf]

AF312688.1

pCLP

AF312688.1

AP020327.1

CP065286.1

JQ657806.1

LT703506.1

CP045326.1

JQ657806.1

CP083742.1

CP079871.1

AP022607.1

AP026368.1

EU271968.1

CP122997.1

LT703507.1

AP022602.1\*

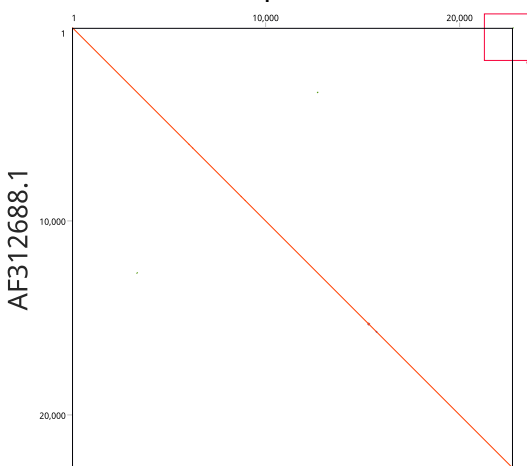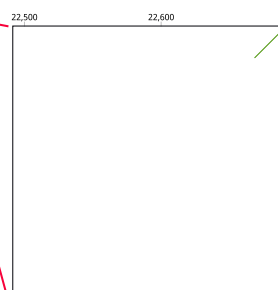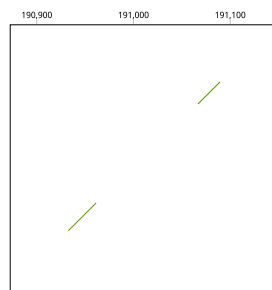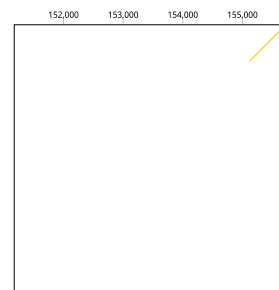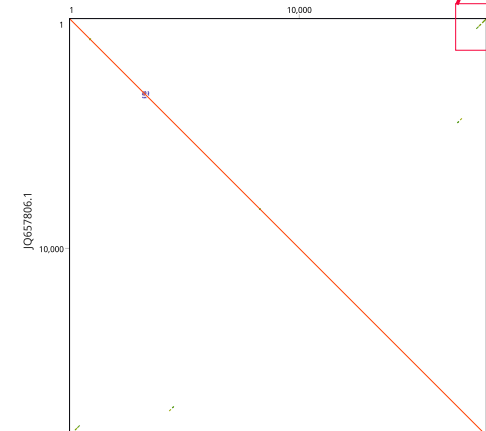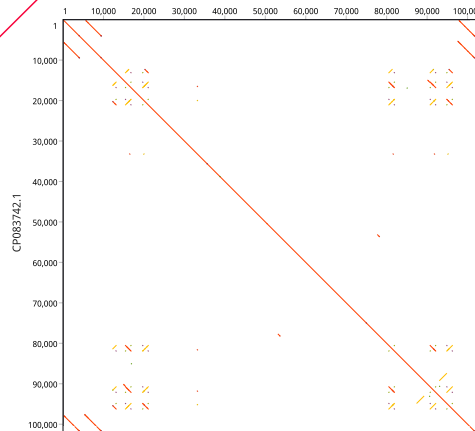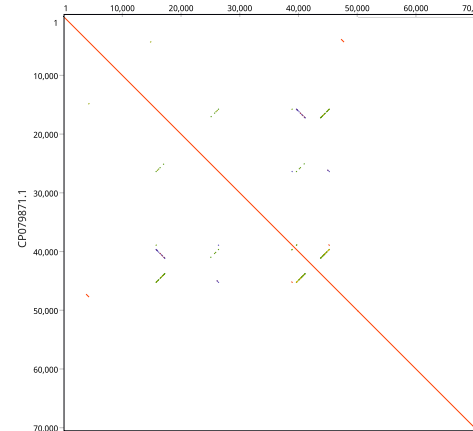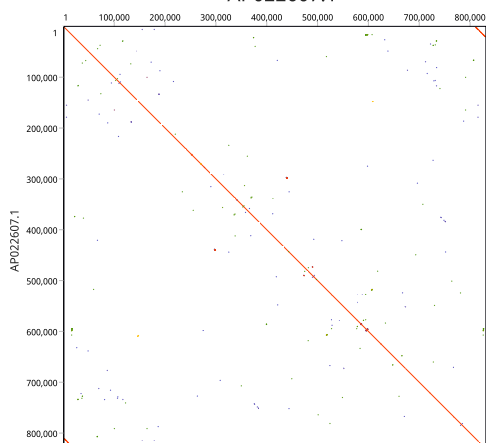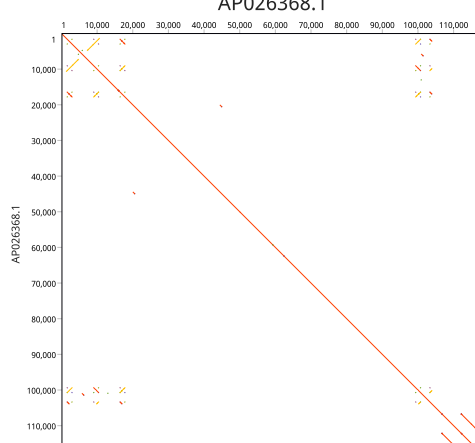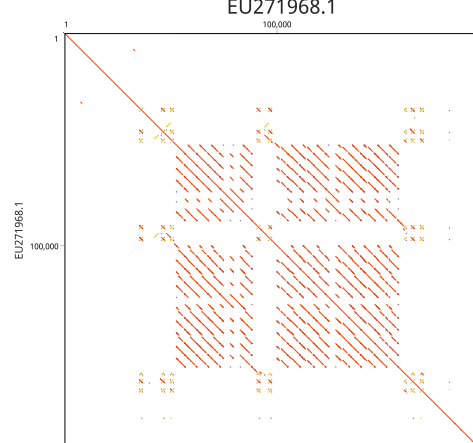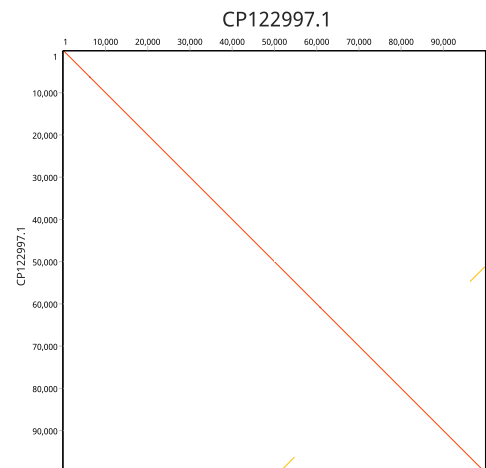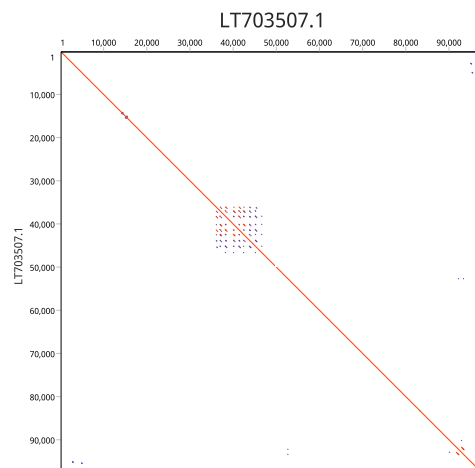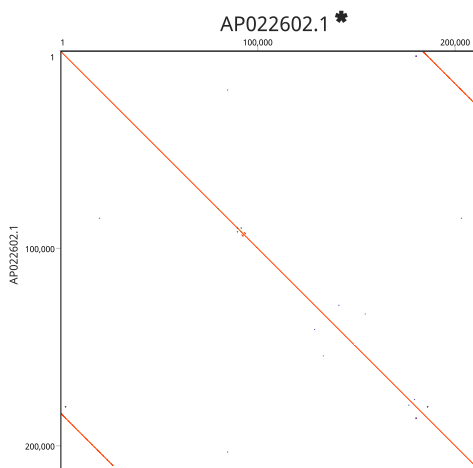

Supplement: Supplementary file 2 — Additional file 2. Supplementary Figures. This file contains all Supplementary Figures and their corresponding legends. [file 13073_2025_1443_MOESM2_ESM.zip › Additional file 2/Fig S2_Dotplots_linearPlasmids.pdf]

Tree scale: 0.1

Plasmid cluster (ID)

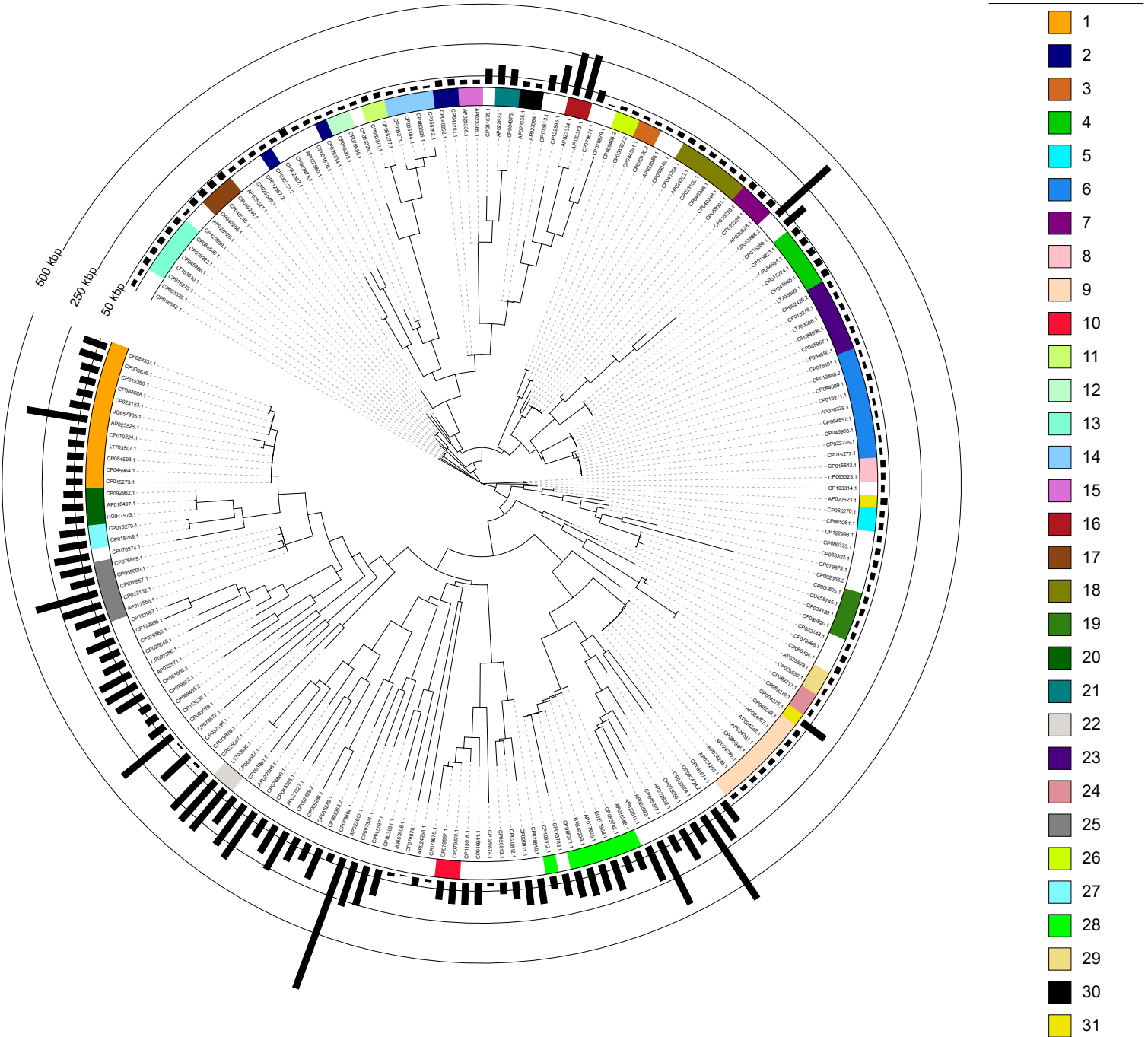

Supplement: Supplementary file 2 — Additional file 2. Supplementary Figures. This file contains all Supplementary Figures and their corresponding legends. [file 13073_2025_1443_MOESM2_ESM.zip › Additional file 2/Fig S3_196plasmidsMashtree_clusters.pdf]

A

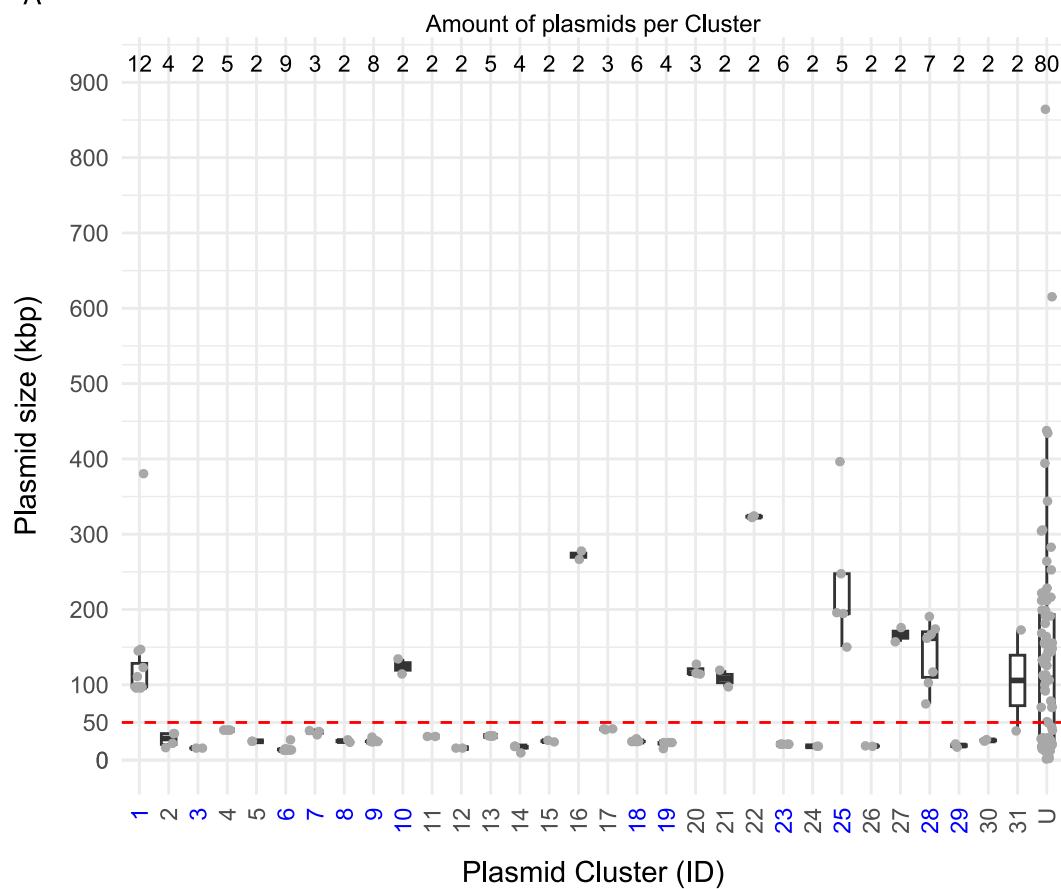

B

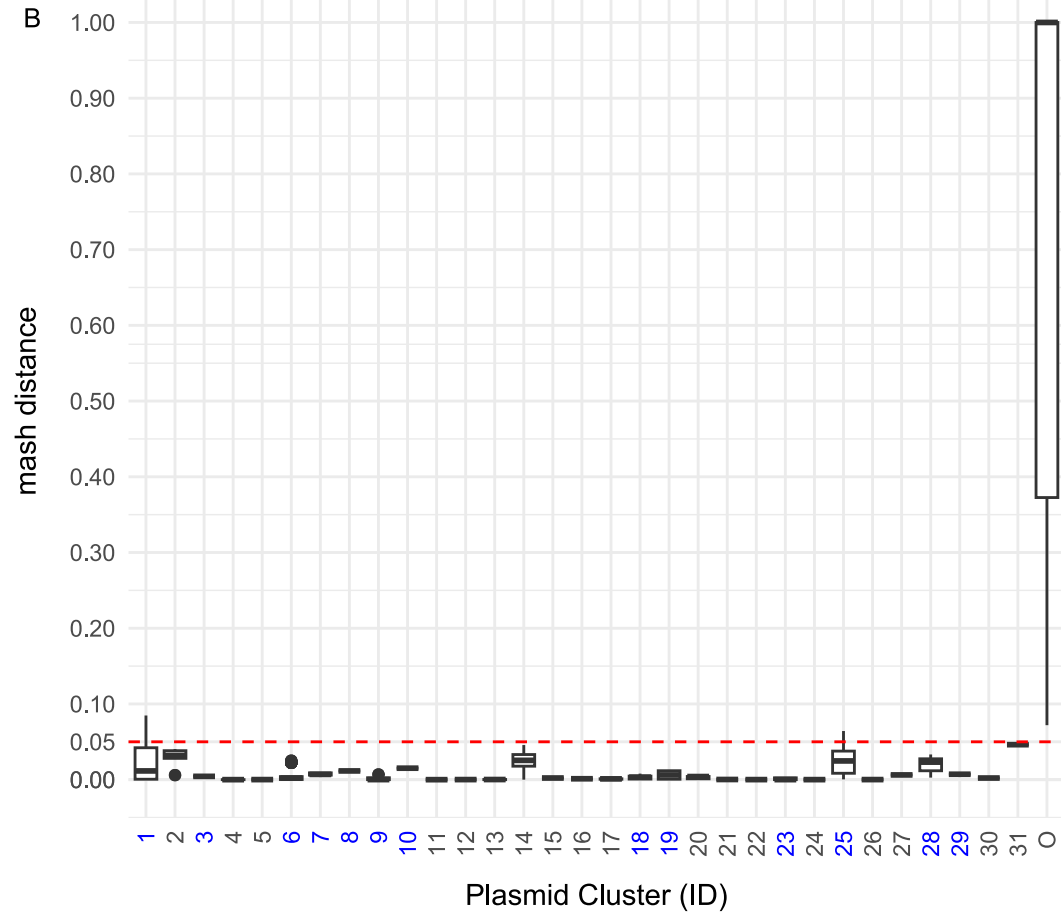

C

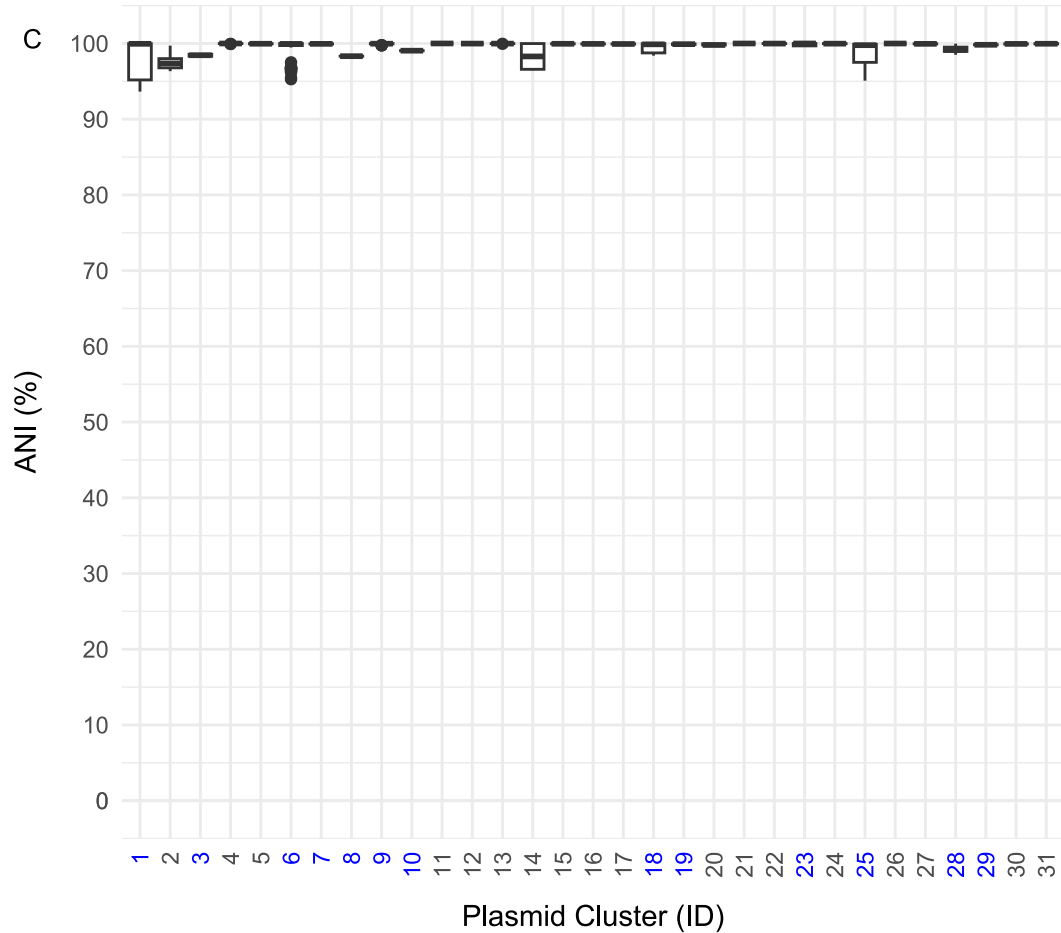

D

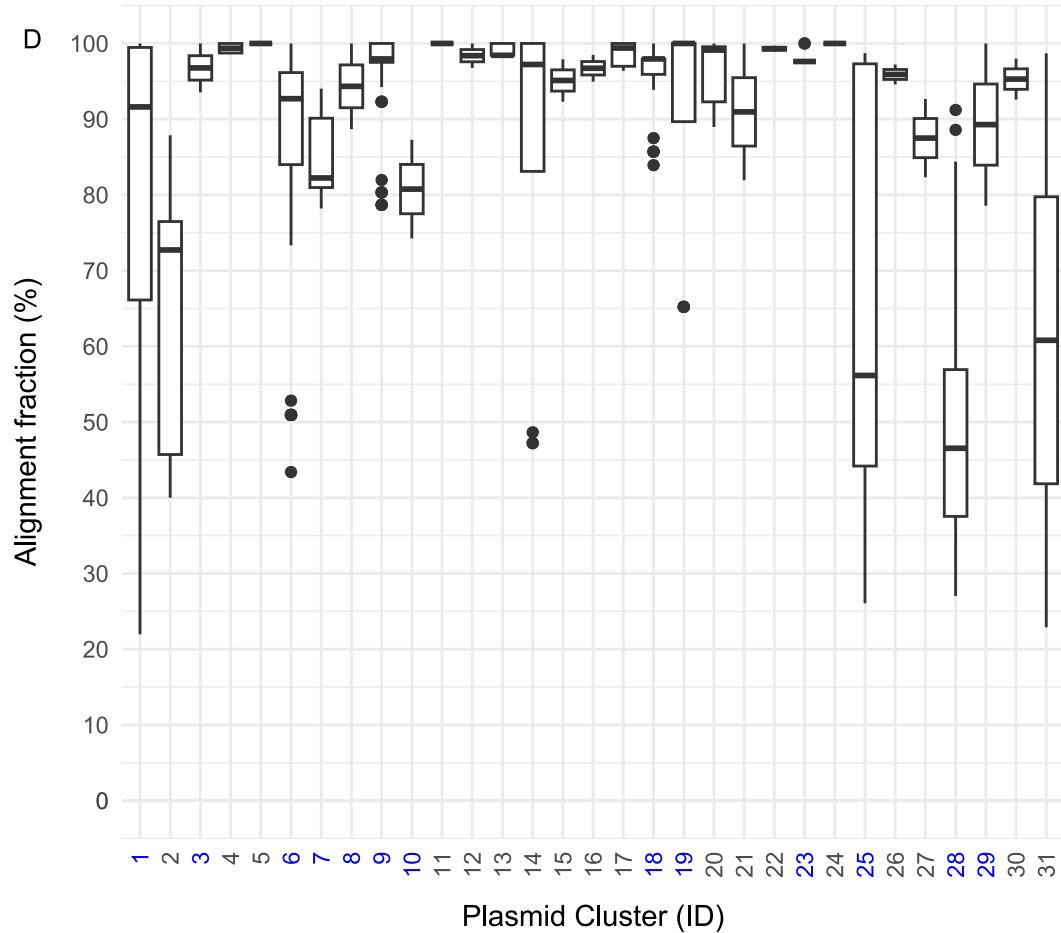

Supplement: Supplementary file 2 — Additional file 2. Supplementary Figures. This file contains all Supplementary Figures and their corresponding legends. [file 13073_2025_1443_MOESM2_ESM.zip › Additional file 2/Fig S4_Characteristics_plasmidClusters.pdf]

A

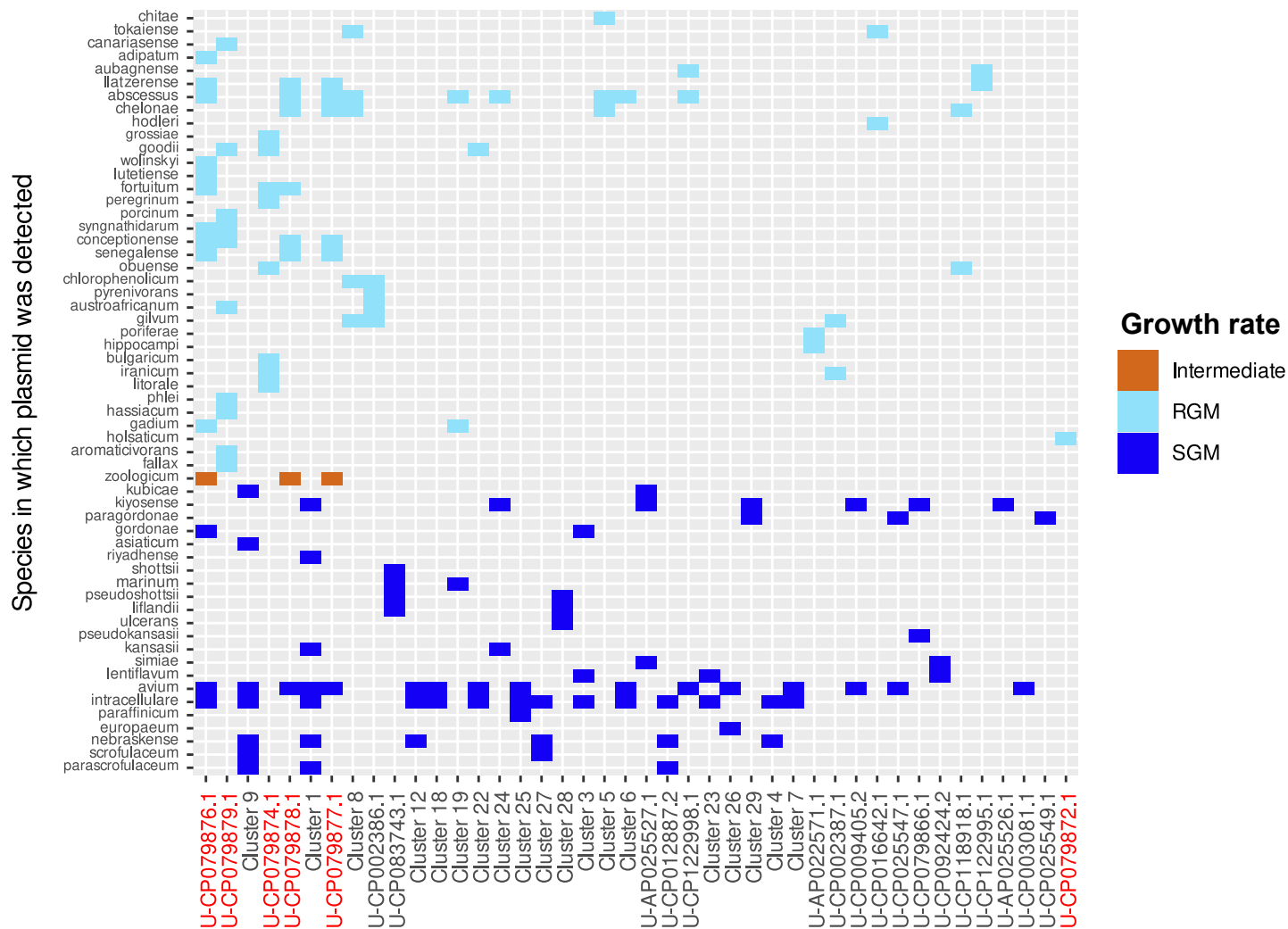

B

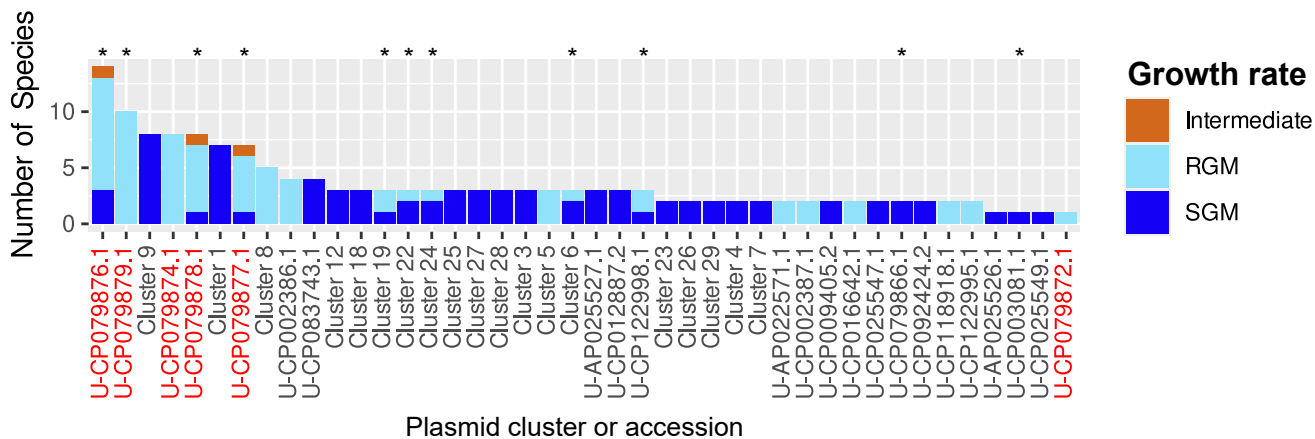

Supplement: Supplementary file 2 — Additional file 2. Supplementary Figures. This file contains all Supplementary Figures and their corresponding legends. [file 13073_2025_1443_MOESM2_ESM.zip › Additional file 2/Fig S5_AbsencePresenceInDraftGenomes.pdf]

A

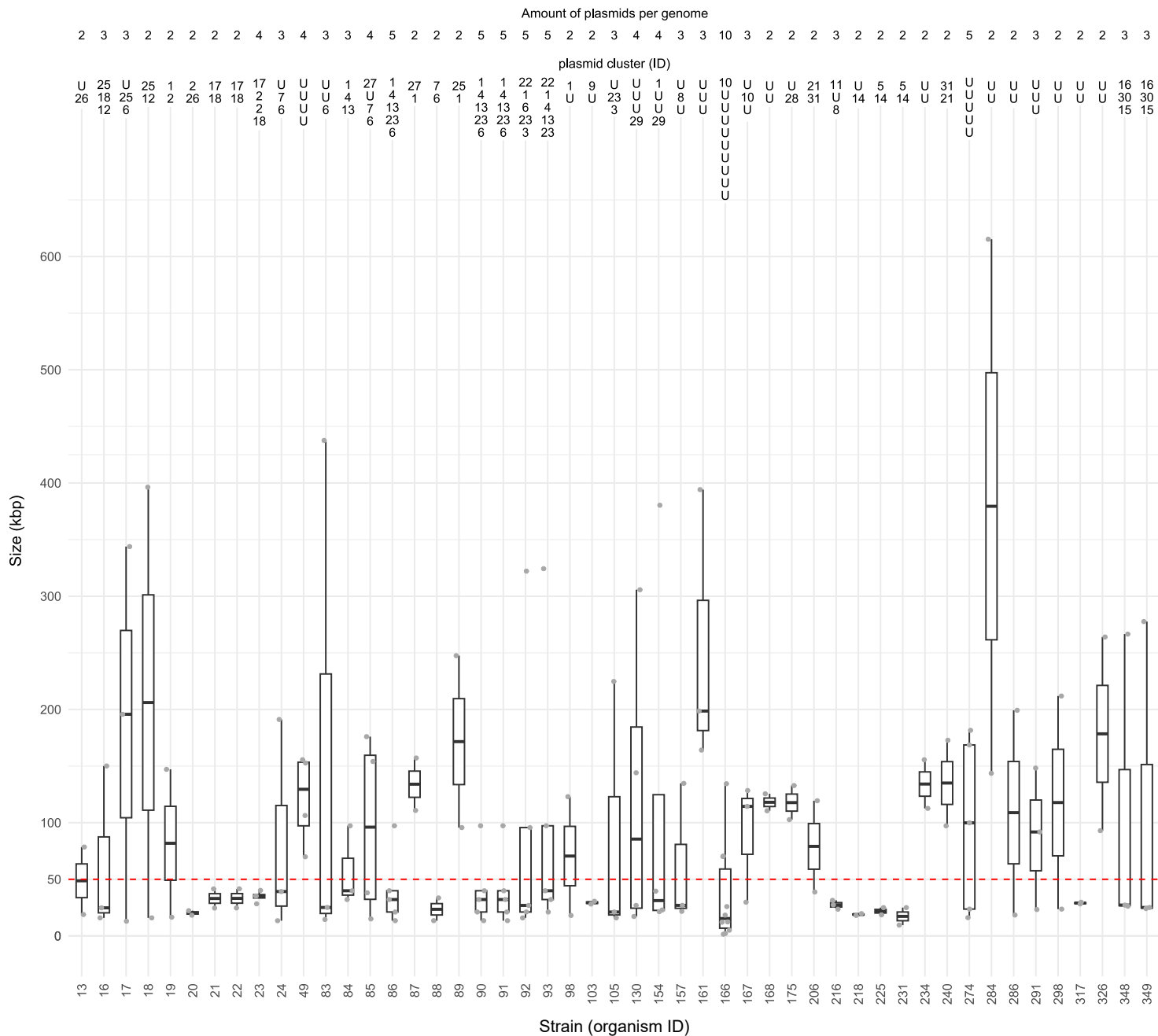

# B

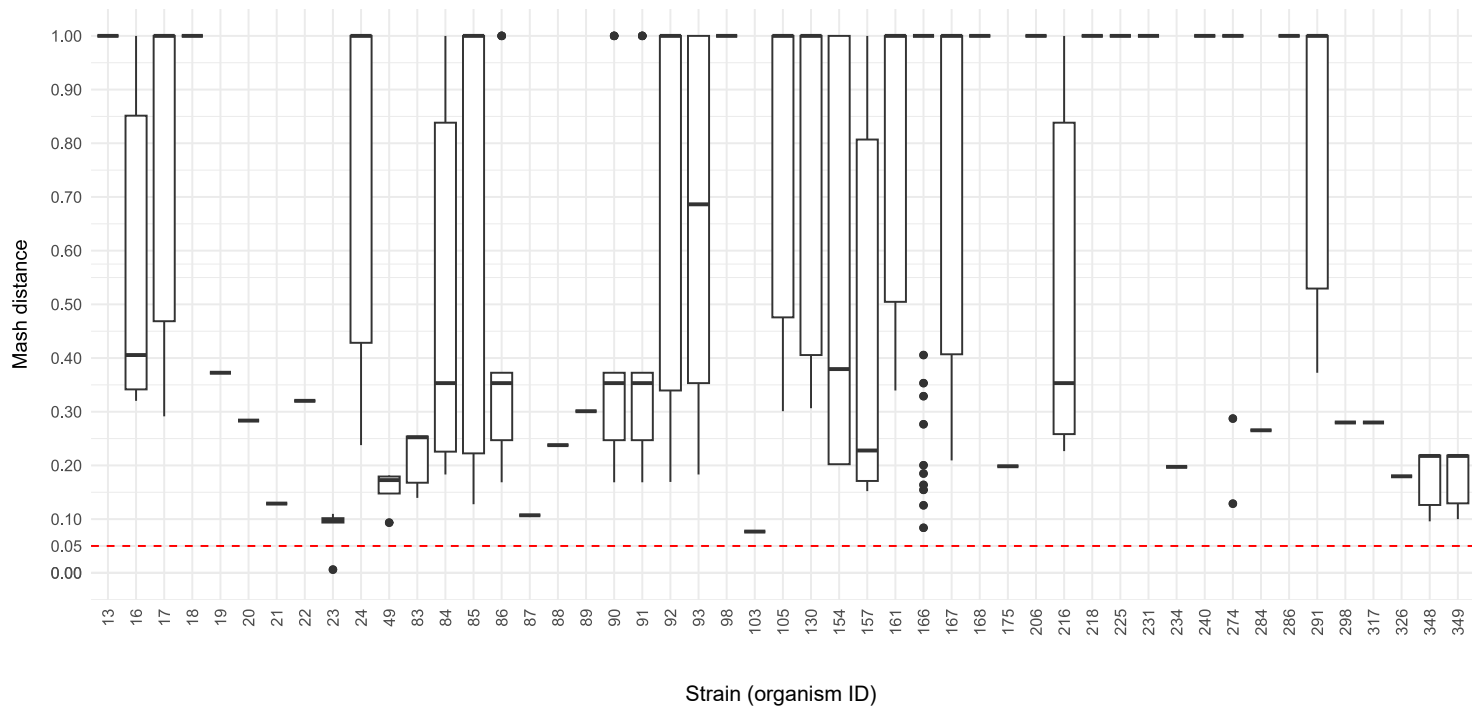

Supplement: Supplementary file 2 — Additional file 2. Supplementary Figures. This file contains all Supplementary Figures and their corresponding legends. [file 13073_2025_1443_MOESM2_ESM.zip › Additional file 2/Fig S6_Characteristics_multi-plasmid-genomes.pdf]

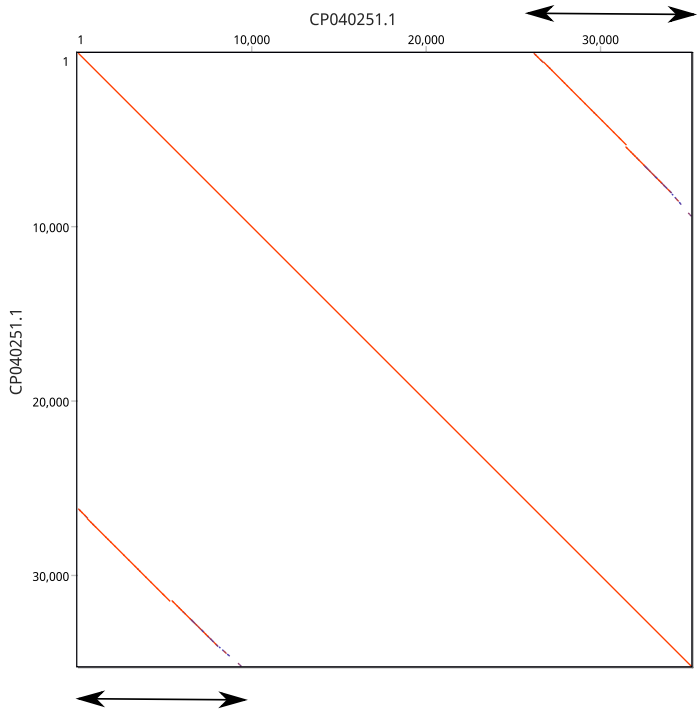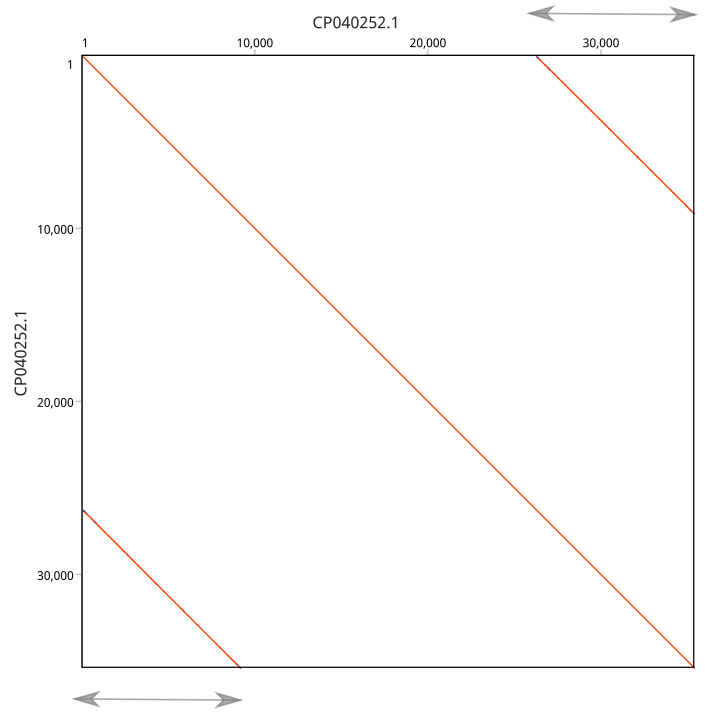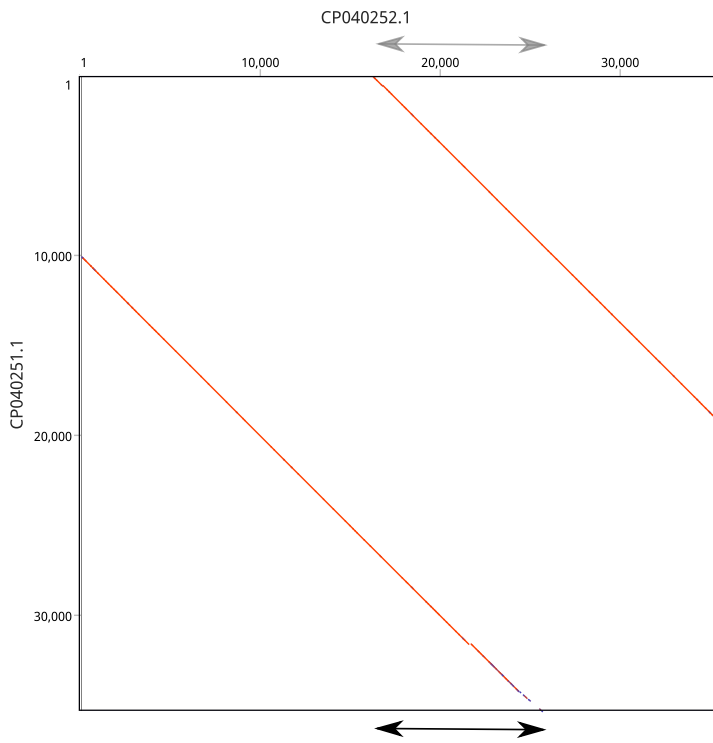

Supplement: Supplementary file 2 — Additional file 2. Supplementary Figures. This file contains all Supplementary Figures and their corresponding legends. [file 13073_2025_1443_MOESM2_ESM.zip › Additional file 2/Fig S7_Dotplots_CP040251-CP040252.pdf]

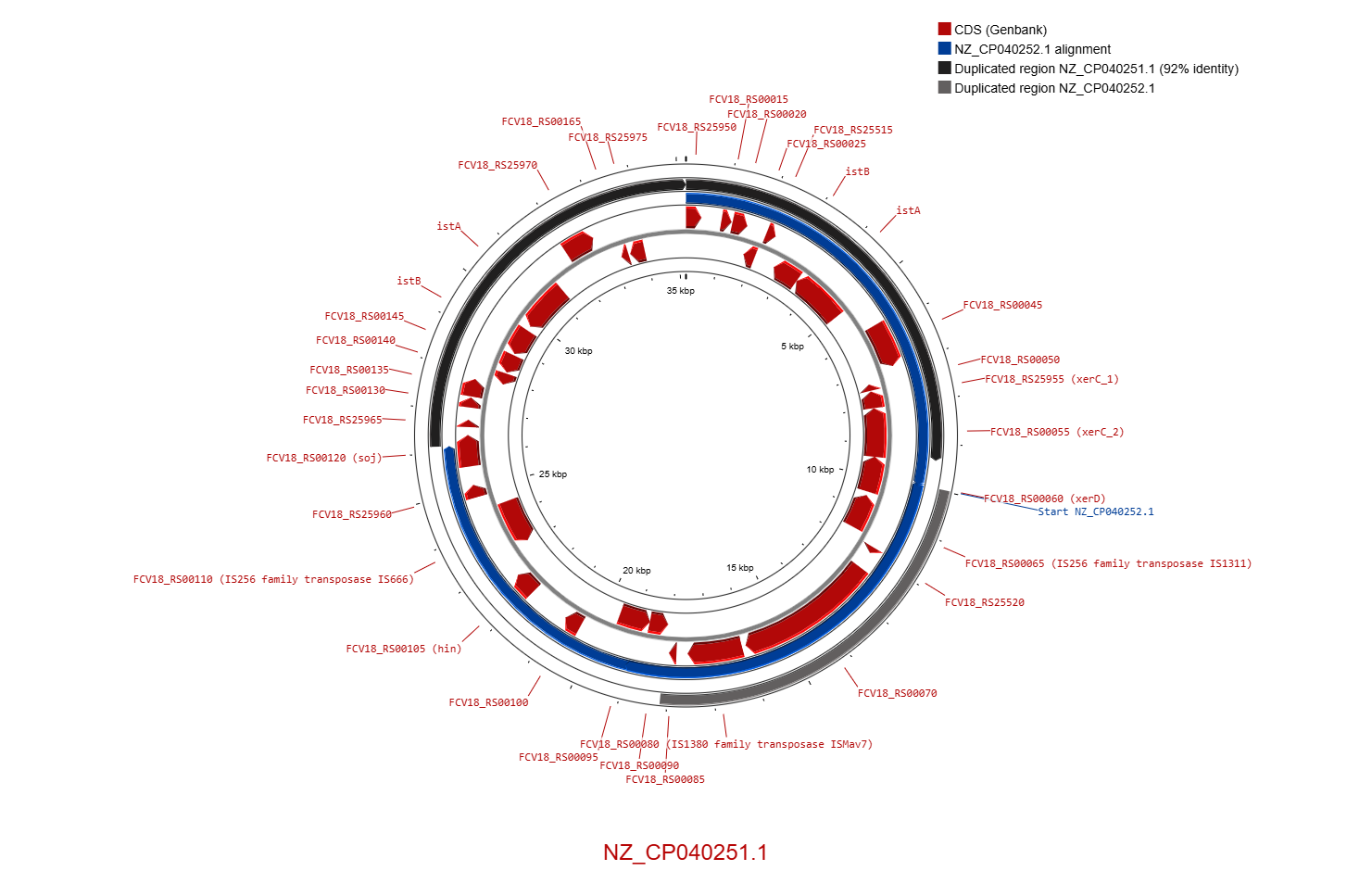

Supplement: Supplementary file 2 — Additional file 2. Supplementary Figures. This file contains all Supplementary Figures and their corresponding legends. [file 13073_2025_1443_MOESM2_ESM.zip › Additional file 2/Fig S8_proksee_CP040251.png]

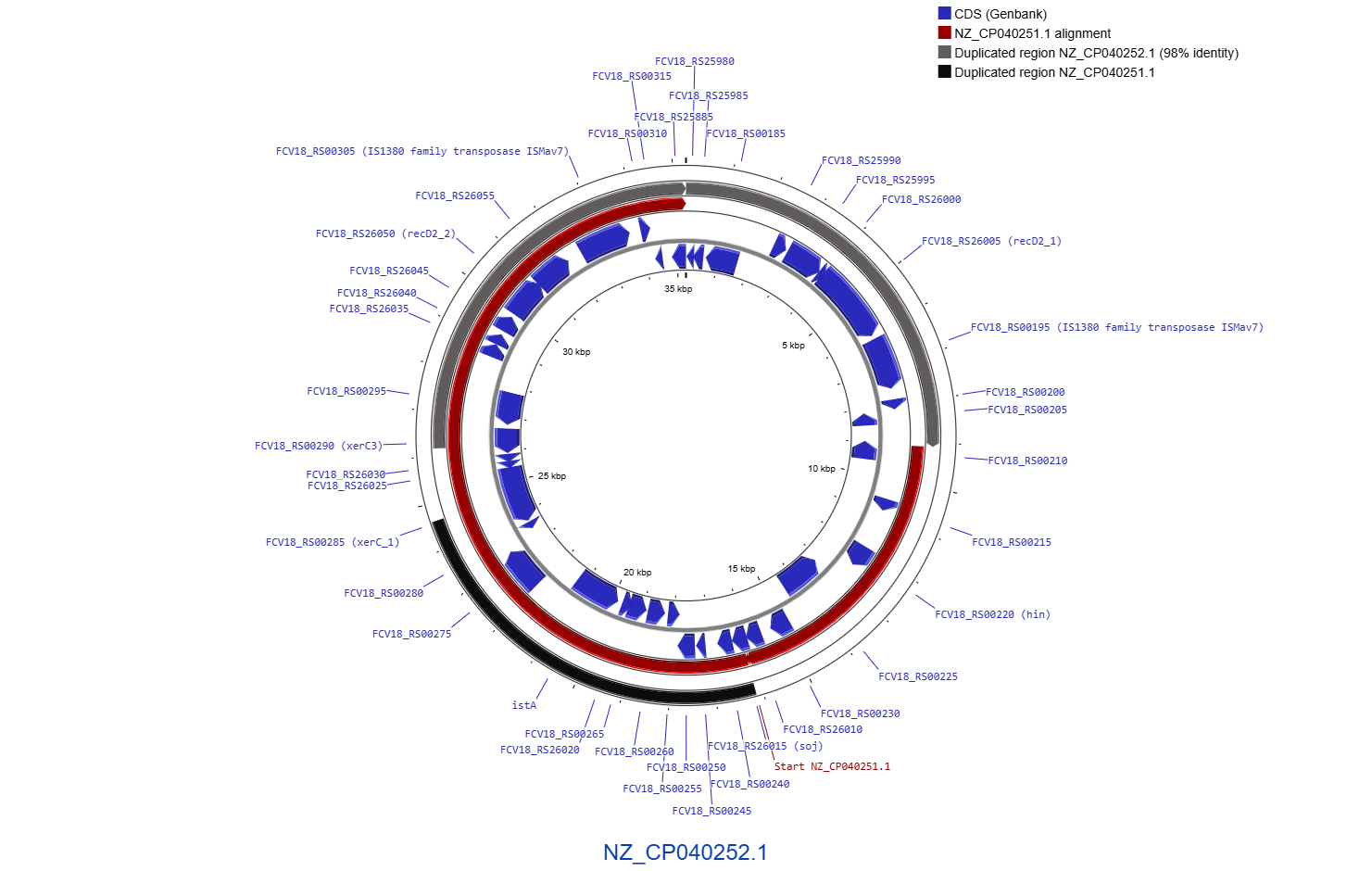

Supplement: Supplementary file 2 — Additional file 2. Supplementary Figures. This file contains all Supplementary Figures and their corresponding legends. [file 13073_2025_1443_MOESM2_ESM.zip › Additional file 2/Fig S9_proksee_CP040252.png]
